# Environmental and social determinants of population vulnerability to Zika virus emergence at the local scale

**DOI:** 10.1186/s13071-018-2867-8

**Published:** 2018-05-08

**Authors:** Erin E. Rees, Tatiana Petukhova, Mariola Mascarenhas, Yann Pelcat, Nicholas H. Ogden

**Affiliations:** 1 Public Health Risk Sciences Division, National Microbiology Laboratory, Public Health Agency of Canada, Saint-Hyacinthe, Québec Canada; 2 Public Health Risk Sciences Division, National Microbiology Laboratory, Public Health Agency of Canada, Guelph, Ontario Canada

**Keywords:** Zika, Environmental determinants, Public health surveillance, Social bias, Risk analysis, Logistic regression model, Accelerated failure time survival model, Colombia

## Abstract

**Background:**

Zika virus (ZIKV) spread rapidly in the Americas in 2015. Targeting effective public health interventions for inhabitants of, and travellers to and from, affected countries depends on understanding the risk of ZIKV emergence (and re-emergence) at the local scale. We explore the extent to which environmental, social and neighbourhood disease intensity variables influenced emergence dynamics. Our objective was to characterise population vulnerability given the potential for sustained autochthonous ZIKV transmission and the timing of emergence. Logistic regression models estimated the probability of reporting at least one case of ZIKV in a given municipality over the course of the study period as an indicator for sustained transmission; while accelerated failure time (AFT) survival models estimated the time to a first reported case of ZIKV in week *t* for a given municipality as an indicator for timing of emergence.

**Results:**

Sustained autochthonous ZIKV transmission was best described at the temporal scale of the study period (almost one year), such that high levels of study period precipitation and low mean study period temperature reduced the probability. Timing of ZIKV emergence was best described at the weekly scale for precipitation in that high precipitation in the current week delayed reporting. Both modelling approaches detected an effect of high poverty on reducing/slowing case detection, especially when inter-municipal road connectivity was low. We also found that proximity to municipalities reporting ZIKV had an effect to reduce timing of emergence when located, on average, less than 100 km away.

**Conclusions:**

The different modelling approaches help distinguish between large temporal scale factors driving vector habitat suitability and short temporal scale factors affecting the speed of spread. We find evidence for inter-municipal movements of infected people as a local-scale driver of spatial spread. The negative association with poverty suggests reduced case reporting in poorer areas. Overall, relatively simplistic models may be able to predict the vulnerability of populations to autochthonous ZIKV transmission at the local scale.

**Electronic supplementary material:**

The online version of this article (10.1186/s13071-018-2867-8) contains supplementary material, which is available to authorized users.

## Background

Zika virus (ZIKV) has been endemic to Africa and Asia for decades [1]. About 20% of infected people show clinical manifestations, which are mostly mild (rash, low grade fever, headache, conjunctivitis and myalgia [2]). Severe complications including Guillain-Barré syndrome [3, 4] and microcephaly and other presumed ZIKV-related birth defects in children born to mothers infected during pregnancy have been reported [5]. The main mechanism of ZIKV transmission is thought to be through *Aedes* mosquitoes (particularly *Ae. aegypti* and *Ae. albopictus*), although sexual and perinatal transmission and transmission by blood transfusion also occur [6–8].

During the new millennium the Asian strain of ZIKV began spreading beyond its traditional range across the Pacific Ocean. In April 2007 an outbreak was detected on Yap Island, followed by Guam and Micronesia. From 2013 to 2014, ZIKV had spread to other Pacific islands including French Polynesia, New Caledonia, Cook Islands, Tahiti and Easter Island [1]. The first autochthonous outbreak on mainland South America was detected by May 2015 in Brazil [9]. However, estimates using genetic methods date the Brazil outbreak as starting sometime between May to December 2013 [1, 10]. From Brazil, ZIKV spread rapidly in South and Central America and the Caribbean. By February 2016, the World Health Organization declared ZIKV a public health emergency of international concern. Autochthonous transmission of ZIKV is now reported throughout South America except Chile and Uruguay as of January 4th, 2018 (http://ais.paho.org/phip/viz/ed_zika_epicurve.asp).

It is not well understood why ZIKV spread rapidly across the Americas following years of relative endemic stability in Africa and Asia. At the international level, a dynamic model of ZIKV transmission in the Americas indicated that spread dynamics were driven by factors affecting mosquito vector occurrence, abundance and activity, and human population characteristics such as international mobility and social status [11]. Abundance and activity of vectors depends on climatic and other environmental factors. Precipitation influences the availability of breeding habitat and *Aedes* spp. abundance is usually higher in wet seasons [12]. Temperature directly influences vector mortality rates and activity, ZIKV development rates within mosquitoes, and indirectly affects vector abundance *via* influences on interstadial development rates. *Aedes* spp. vectors of ZIKV (*Ae. aegypti* and *Ae. albopictus*) have an approximate range of temperatures suitable for reproduction and survival of at 16–35 °C [13]. Higher temperatures shorten the duration of the extrinsic incubation period enabling faster rates of transmission [14]. Elevation is correlated with temperature (higher elevations being cooler) but may also be a proxy for different habitats that have more complex interactions with mosquito survival and reproduction [15].

Human social factors may affect exposure to infected vectors and also influence bias in case detection by surveillance systems. Detection of ZIKV may be more likely in dense populations because there may be more availability of healthcare facilities. Poverty is associated with higher rates of transmission of *Aedes*-borne pathogens amongst humans [16]. Poorer areas have lower quality housing, with lack of window screens and often more peridomestic *Aedes* spp*.* aquatic breeding habitat leading to higher mosquito abundance and biting rates [17, 18]. However, poorer populations may have less access to healthcare and education for awareness and prevention, resulting in lower disease detection by surveillance programs [19, 20]. Finally, rates of population movement will determine rates of spread of infection into pathogen-free areas [1, 21], and these rates can be influenced by poverty [22].

Clearly the potential for, and speed of, ZIKV spread depends on the abundance and activity of vectors [11, 23], the level of human exposure and human density, global trade and human travel [11, 24], to some extent sexual transmission [8], and potentially the level of immunity in the human population [25]. Given that there is no clear evidence for ZIKV immunity [26, 27], it is likely that naive human population immunity in South and Central America combined with high abundance of vectors and suitable conditions for transmission and spread contributed to the rapid spread of ZIKV, as observed with the emergence and spread of chikungunya in the region [28]. The few areas not reporting the disease likely had either habitat inhospitable for vectors (e.g. alpine regions) or were missed by the surveillance system.

The objective of our study was to characterize population vulnerability to sustained local autochthonous transmission of ZIKV as derived from environmental, social and neighbourhood disease intensity factors. We used two modelling approaches on the same surveillance dataset to concentrate on the potential for autochthonous transmission versus the timing of emergence. Our analysis focused on Colombia, which suffered the second largest outbreak after Brazil. We discuss the implications of our dual-modelling approach for risk analysis in the context of advising effective public health management of ZIKV.

## Methods

### Study area

Colombia is geographically diverse with elevation ranging from sea level to > 5000 m in the Andes, and there are 11 Köppen climate zones [29]. In general, areas below 1000 m are hot (> 24 °C), 1000–3000 m are temperate (12–24 °C) and areas above 4000 m are typically below 12 °C and often experience sub-zero temperatures. The climate is suitable for *Aedes* species and ZIKV transmission, except at high elevations [30, 31]. Most Colombians live at mid-altitudes in the temperate zones. Colombia is a developing country and while measures of health, education and wealth are improving, there still remains significant levels of poverty particularly in low-lying and rural areas [32].

### Surveillance data

ZIKV cases included all patients who reported symptoms to surveillance services of Instituto Nacional de Salud (INS), Colombian Government. Cases were then classified as suspect or confirmed cases given the INS case definitions (for details see Additional file 1). In brief, suspected cases were individuals presenting with a rash and fever along with one or more clinical symptoms consisting of non-purulent conjunctivitis, headache, pain in body or joints, or edema in lower extremities. The suspected case definition also included the criteria that the individual has come from or visited places in Colombia below 2200 m in elevation and with confirmed circulation of ZIKV at least 15 days prior to the onset of symptoms, and/or countries with or without confirmed circulation of ZIKV. Confirmed cases were individuals who presented with clinical symptoms of ZIKV disease and tested positive for ZIKV in laboratories affiliated with the National Network of Laboratories, National Institute of Health, or collaborating centers designated by the INS. Confirmed cases also included a case definition typically associated with probable cases to describe individuals with clinical symptoms as described above, who had visited sites with elevations less than 2200 m in Colombia with confirmed circulation of ZIKV in the 15 days prior to the onset of symptoms. Laboratory confirmed cases were cases with a positive result for ZIKV by RT-PCR or by serological immunoassay testing [9, 33]. The case date for a patient was the week that they had presented themselves for care at health centres [9].

In Colombia, the first case of ZIKV was detected in the epidemiological week (EW) 41 in October of 2015 [9, 34]. Cases have since been confirmed back to EW 32 (August) of 2015 [35], and may have occurred as early as April [11]. The Colombian surveillance system has collected data on ZIKV cases at the municipal level on a weekly basis since January 9th 2016 (http://www.ins.gov.co), and these are the data we used for our study up to the third week in September 2016. To contend with missing data from 2015, we first grouped suspected with confirmed case data at the municipal and week levels. Secondly, if the number of reported cases was greater than one when first reported, we back-estimated the likely date of the first case for that municipality by assuming an exponential growth rate to the power two [36]. For example, if 8 cases were reported at the first week, *t* = 0, of surveillance on January 9th 2016, then there were 4 cases at week *t*-1, 2 cases at week *t*-2, and 1 cases at week *t*-3.

To visually inspect for spatial patterns in reported emergence at the municipal level for the mainland we mapped days to first report in ArcGIS Map v. 10.5 (www.esri.com) using a GIS layer obtained from the GADM database, version 2.8 (www.gadm.org).

### Statistical models

**Fig. 1:**
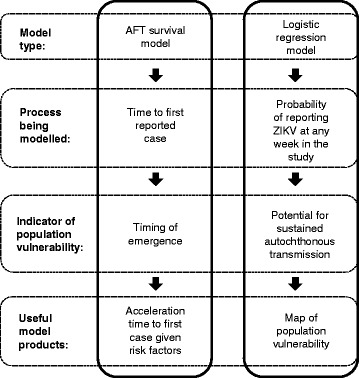
Summary of modelling approach using logistic regression and AFT survival models

An overview of the modelling approach is summarised in Fig. 1 and Table 1. We used logistic regression that accounted for serial dependence in the longitudinal surveillance data and survival modelling approaches. It is important to account for serial dependence to ensure model output is not misleading given the assumption of independent observations [37]. For details on model formation and assumptions see Additional file 2, but briefly, the logistic regression was used to estimate the probability of ZIKV being reported in a municipality *i* at week *t*. Except for the municipality with the index case for the study area, at the start of the study period the outcome was negative, y = 0, and remained at y = 0 for each *t* until at least 1 ZIKV case was detected, changing the outcome to y = 1. If an outcome was detected, the outcome remained at y = 1 until the end of the study period (Table 2). We consider this a valid assumption given that most municipalities reporting ZIKV had epidemiological incidence curves typical of infectious diseases (i.e. increase, peak, and decrease in cases over time). Furthermore, almost 99% of these municipalities continued reporting ZIKV by the last week in the study period.

**Table 1:** Summary of modelling approach given variables, their structure and hypothesized effects

Model variables		Temporal scale	Municipal-level observations at temporal scale for *n* weeks of the outcome variable given model type and data structure defined in Table 2	Hypothesized effect
Environmental	Mean study period temperature, *Ts* (°C)	Study period	*Ts*, *Ts*, *Ts*, …*Ts*	Vector habitat suitability
	Mean weekly daytime temperature, *Td* (°C)	Weekly	*Td*_*1*_, *Td*_*2*_, *Td*_*3*_, …*Td*_*n*_	Vector reproductive and survival rates; Viral extrinsic incubation period (EIP)
	Mean weekly nighttime temperature, *Tn* (°C)	Weekly	*Tn*_*1*_, *Tn*_*2*_, *Tn*_*3*_, …*Tn*_*n*_	Vector reproductive and survival rates; Viral generation time
	Total study period precipitation (mm), *Ps*	Study period	*Ps*, *Ps*, *Ps*, …*Ps*	Vector habitat suitability
	Total weekly precipitation (mm), *Pw*	Weekly	*Pw*_*1*_ *, Pw*_*2*_ *, Pw*_*3*_ *, …Pw*_*n*_	Vector reproductive rate
	Mean Elevation (m), *E*	Study period	*E*, *E*, *E*, …*E*	Vector reproductive and survival rates; EIP
	Mean vector environmental suitability, *V*	Study period	*V*, *V*, *V*, …*V*	Vector habitat suitability; Vector reproductive and survival rates; EIP; Human exposure in urban versus rural areas
Social	Population density per km^2^, *D*	Study period	*D*, *D*, *D*, …*D*	Detection (higher population linked to more case investigations)
	Unsatisfied Basic Needs (% population), *U*	Study period	*U*, *U*, *U*, …*U*	Exposure related to housing quality; Access to health centres
	Inter-municipal road connectivity, *Rc*	Study period	*Rc*, *Rc*, *Rc*, …*Rc*	Local movement of people to spread ZIKV
	Road density per km^2^, *Rd*	Study period	*Rd*, *Rd*, *Rd*, …*Rd*	Local movement of people to spread ZIKV
Neighbourhood disease intensity	Nearest infected municipality (km), *Nn*	Weekly	*Nn*_*1*_, *Nn*_*2*_, *Nn*_*3*_, …*Nn*_*n*_	Controls for areas with autochthonous ZIKV transmission
	Proportion of neighbouring municipalities reporting ZIKV, *Np*	Weekly	*Np*_*1*_, *Np*_*2*_, *Np*_*3*_, …*Np*_*n*_	Controls for areas with autochthonous ZIKV transmission

**Table 2:** Example structure of the serial outcome variables used in the logistic regression and the accelerated failure time (AFT) models as derived from the 48 week ZIKV surveillance data for a positive outcome (y = 1), negative outcome (y = 0), or right-censored data

Modelling approach	Possible outcome, y	Weeks of study period							
		1	2	3	4	5	6	…	48
Logistic regression	Index case	1	1	1	1	1	1	…	1
	First case detected in week 3	0	0	1	1	1	1	…	1
	No cases detected	0	0	0	0	0	0	…	0
AFT model	Index case	1	–	–	–	–	–	…	–
	First case detected in week 3	0	0	1	–	–	–	…	–
	No cases detected	0	0	0	0	0	0	…	0

A survival regression model was used to estimate the time *t* to report a first case of ZIKV in municipality *i*, under the assumption that all municipalities can experience the event of reporting ZIKV. We adopted a parametric technique to efficiently use all of the information we had about the observations. Assuming that the presence of the first reported case of Zika accelerates the infection growth rate exponentially and then taking into consideration that the number of cases decays within a timeframe due to different processes [36], we modeled the time to the first report of ZIKV in municipalities with an accelerated failure time (AFT) model. Results from the AFT model can be used to quantify the role of variables in accelerating or slowing the time to the event (i.e. first reported case at week *t*). The distributional form of the baseline hazard was assessed using diagnostic procedures based on Akaike’s information criterion (AIC) and from graphical examination. The effect of time-dependent variables to influence the hazard over time was accommodated by including variable observations at week *t* from *t* = 0 up to the week of the first reported case as formatted for right-censored data (Table 2).

### Explanatory variables

For details on explanatory variables see Additional file 2, but briefly, environmental variables explored were those that are known determinants of vector abundance and activity, as well as ZIKV development rates in mosquitoes. A vector environmental suitability variable came from a previously developed composite measure of *Aedes* spp. vector habitat and arbovirus transmission suitability at a 5 km resolution [31]. Total and weekly study period precipitation variables were derived at 0.1 degree latitude/longitude resolution from Integrated Multi-satellitE Retrievals for GPM (IMERG) from NASA [38]. Study period and weekly mean surface daytime and nighttime temperature variables were derived at 0.05 degree latitude/longitude resolution from Moderate Resolution Imaging Spectroradiometer (MODIS) [39]. Elevation data came from a 90 m resolution digital elevation model [40].

Social variables were considered as determinants of ZIKV transmission within and between municipalities. Data for municipal population size were available from the Colombian Government (DANE; http://www.dane.gov.co) and were converted to densities using municipal areas. Our measure of poverty was the percentage of the municipal population with unsatisfied basic needs (UBN). UBN is a common poverty metric for Latin America [32, 41] and was available from the Departamento Administrativo Nacional de Estadística (DANE: http://www.dane.gov.co). Municipal road density was considered a measure of human connectivity within a municipality, and obtained from the Center for International Earth Science Information Network (CIESIN) [42]. We used the same road network dataset to calculate a measure of inter-municipal connectivity as the number of roads entering/leaving the municipality.

Neighbourhood infection intensity variables controlled for the spatial distribution and temporal progression of cases reported in municipalities. The rate at which municipal populations become infected likely depends on their proximity to ZIKV-affected areas. We tested two variables to represent proximity to other infected municipalities at time *t* as distance (km) to the nearest infected municipality, and the proportion of infected neighbouring municipalities. These variables were created using the imputed surveillance data, in that, once a municipality had reported ZIKV it was assumed to remain a source of ZIKV over the course of the study period. In the logistic regression models we accounted for the temporal progression of ZIKV by including a categorical variable for week of reporting as a proxy for the growth rate of infection in the population.

### Model building

Missing values in the MODIS temperature data (approximately 10%) were imputed using a two-step approach (see Additional file 2). For the logistic model, the variables were centred by their mean and then scaled to remove collinearity and to ensure that the regression coefficient estimates were on the same scale. For the AFT model, the variables were rescaled, as appropriate, to have meaningful baseline hazards by subtracting the minimum value from each observation. We derived the functional form of the variables with the response variable of the respective model and verified that they did not violate model assumptions of linearity. We examined all pairwise correlations among predictor variables. Highly correlated variables (Pearson’s *r* ≥ 0.5) were not included in the same multivariable model.

We built multivariable models to investigate the role of environmental, social and neighbourhood disease intensity variables. This included assessing for different temporal scales of temperature and precipitation data (e.g. study period mean, current and weekly time lags) Also, we tested for interacting effects between poverty and each of our connectivity metrics which could indicate that underreporting in poorer areas results from less road infrastructure and/or reduced mobility of infected people into these areas.

### Model selection and validation

We selected our best logistic and AFT models using a forward stepwise process given the criteria of retaining statistically significant variables (*P*-values from the t-test and Wald test of the individual coefficients of the logistic and AFT models respectively), minimising AIC and maximising model parsimony (i.e. when competing models were within 2 AIC of each other we selected the more parsimonious model), and conforming to the assumptions of parametric modelling. For the logistic regression we calculated the predictive ability of the best selected model using the receiver operating characteristic (ROC) area under the curve (AUC) in R using package *ROCR*. The AUC value indicated the ability of the model to correctly classify the observed outcomes: 0.9–1.0, excellent; 0.8 to < 0.9, good; 0.7 to < 0.8, fair; 0.6 to < 0.7, poor; 0.5 to < 0.6, fail [43]. The AFT model was validated by verifying that the residuals conformed to the parametric distribution and by using a 10-fold validation method to check model accuracy (Additional file 3).

### Predicting population vulnerability to ZIKV emergence

Our best logistic regression model was used to map population vulnerability to sustained autochthonous transmission of ZIKV as the relative probability of reporting ZIKV given environmental and connectivity variables. This provides an estimate of the general vulnerability irrespective to the space-time progression of the disease and reporting biases associated with poverty. Model predictions were calculated for a hexagonal grid of the median area of Colombia municipalities (275 km^2^) to show population vulnerability at a homogeneous spatial scale. ArcGIS Map v. 10.5 (www.esri.com) was used for all mapping.

To help interpret the effects of variables retained in the best AFT model on the timing of a first case of ZIKV being reported, we plotted the acceleration of time to infection over the range of variable values, while holding constant the other variables at their median value for the study period (except for the proportion of neighbouring municipalities reporting ZIKV, which we held constant at 0.5). We used the acceleration of time-to-the-event to interpret the role of the variable to accelerate or delay the timing of the first case, given the range of variable values. Variable values have an effect to increase time to the first report when the acceleration factor (AF) value is ≥ 1 and decrease time to the first report when the AF is < 1.

## Results

### Surveillance data

**Fig. 2:**
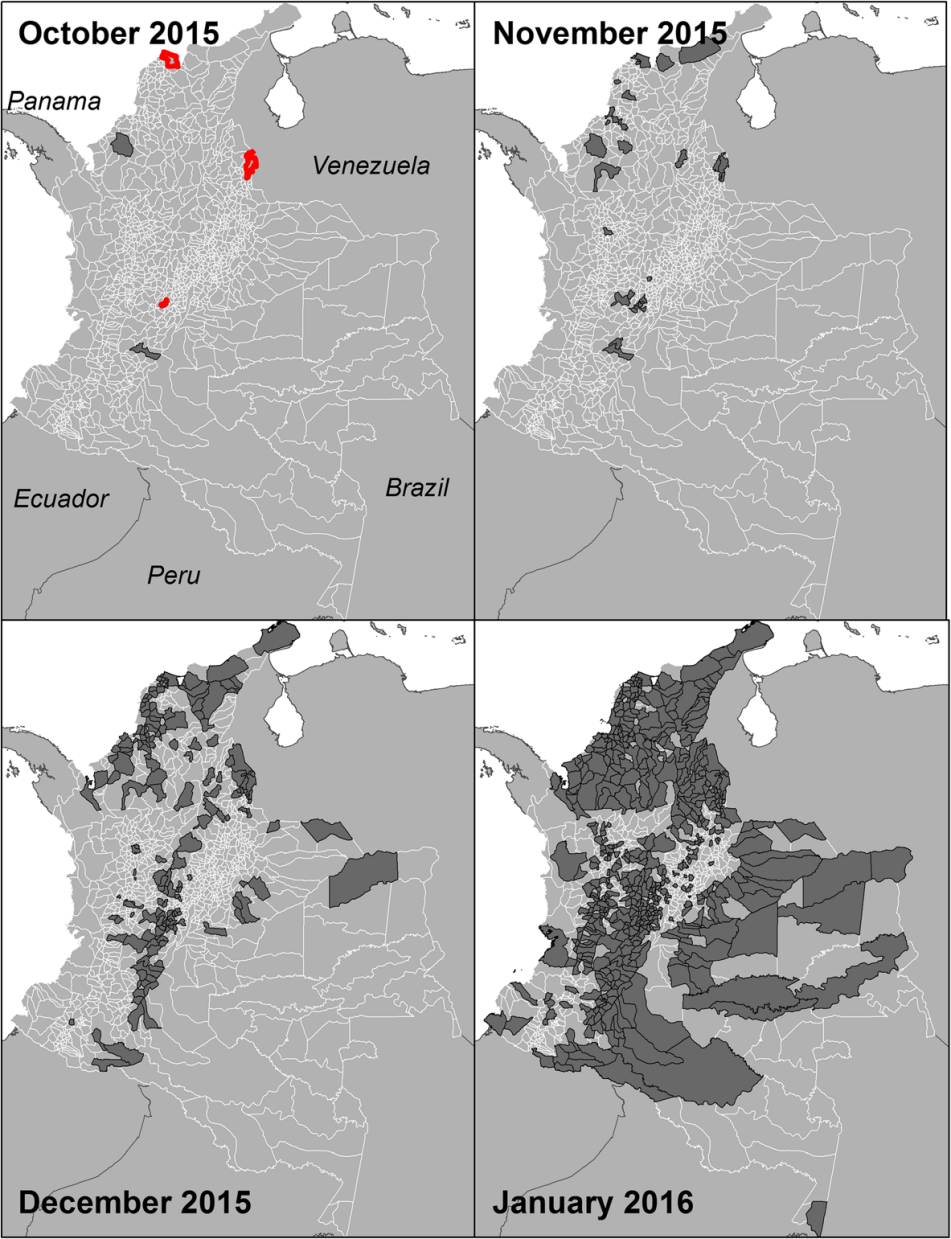
Municipalities reporting ZIKV in Colombia in 2015 up to the end of October, November, and December, as well as January 2016. Municipalities estimated to be earliest case locations for October are outlined in red

**Fig. 3:**
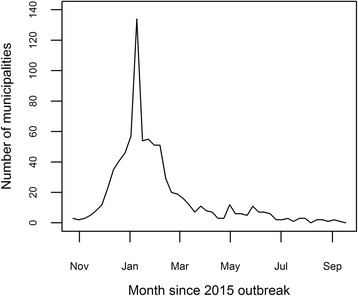
The number of municipalities reporting their first case of Zika, per week, from October 2015 to September 2016

We identified three spatially distinct locations on October 24th 2015 in the municipalities of Barranquilla, Girardot and San José de Cúcuta from backtracking case reports greater than 1 on January 9th 2016 (Fig. 2). We used October 24th 2015 as the start of our study period, which aligns well with a Pan American Health Organization report suggesting that case incidence began growing exponentially by approximately week 39 (i.e. October 2015) [35]. From October 24th 2015, the number of municipalities reporting their first case increased up to a peak in January 2016, and then decreased to approximately 1–2 municipalities per week in the later epidemic phase (Fig. 3). Municipalities with early emergence dates clustered around the early case locations (Fig. 2). The west coast, central-north and south-eastern regions of the country had later emergence dates. There are also notable outliers of municipalities that were late to report (or did not report ZIKV) and were surrounded by municipalities that reported ZIKV early, such as in the northern part of the country by January 2016. Of the 1062 municipalities with surveillance data, 1019 had with complete time series of variable data available for model building. By the end of the study period 797 of 1019 municipalities (78%) had reported a first case of ZIKV.

### Factors affecting probability of ZIKV emergence

The best performing logistic regression model (Table 3) had an AUC = 0.91. The probability of reporting ZIKV increased with warmer mean study period nighttime temperatures, higher inter-municipal connectivity, an increasing proportion of neighbouring municipalities reporting ZIKV, and time. The reporting probability decreased with increasing total study period precipitation, and increasing poverty, especially in areas with lower connectivity (Additional file 3: Figure S3.1.a). See Additional file 3 for details on model output, variable effects and candidate models.

**Table 3:** Parameters of the best model for the probability of reporting a first case of ZIKV in municipality *i* at week *t*

Coefficient	Estimate	SE	*t*-value	*P*-value
Intercept	-12.00	0.97	-12.40	< 0.01
Mean study period temperature	4.09	0.24	16.80	< 0.01
Total study period precipitation	-0.55	0.09	-5.98	0.04
UBN	-0.93	0.32	-2.95	< 0.01
Connectivity	0.95	0.25	3.86	< 0.01
Proportion of neighbours reporting ZIKV	0.14	0.04	3.80	< 0.01
UBN × Connectivity	-0.37	0.16	-2.38	0.02

*Notes*: UBN, unsatisfied basic needs; Connectivity, inter-municipal road connectivity; SE, standard error. See Additional file 3 for full model parameters and definitions of variable functional form transformations

**Fig. 4:**
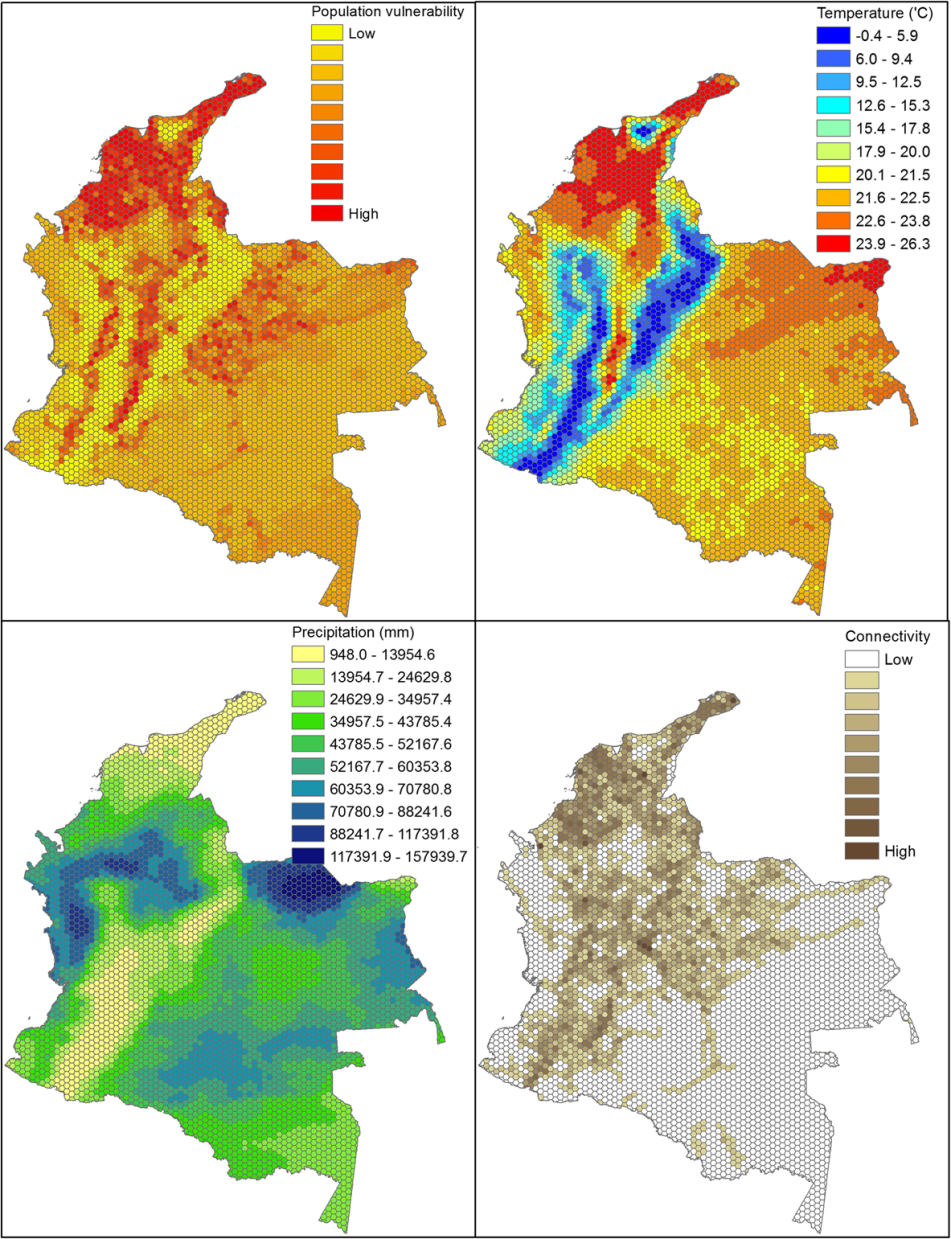
Population vulnerability to ZIKV emergence as derived from the top selected model for the probability of reporting a first ZIKV case. The mapped values for the variables in the top selected model (respectively mean study period nighttime temperature, total study period precipitation and inter-hexagon road connectivity)

Our best model was used to calculate the relative probability of reporting ZIKV to identify populations vulnerable to sustained autochthonous transmission using variables: mean study period nighttime temperature, mean study period total precipitation and inter-municipal connectivity. The variables for UBN and reporting week were not included (i.e. set to 0). The relative probability, as mapped over a hexagon grid, indicates that populations with high vulnerability to ZIKV emergence are clustered and occur in the northern and central regions of Colombia (Fig. 4). In visually assessing for correlations with temperature and precipitation it appears that precipitation is not a limiting factor unless it is low and occurs congruently with low temperature, as occurs in high alpine areas in central Colombia (Fig. 4). Connectivity appears to help facilitate higher vulnerability in areas where temperature and precipitation are not limiting, which is more apparent in the eastern half of the country (Fig. 4).

### Factors affecting time to ZIKV emergence

**Fig. 5:**
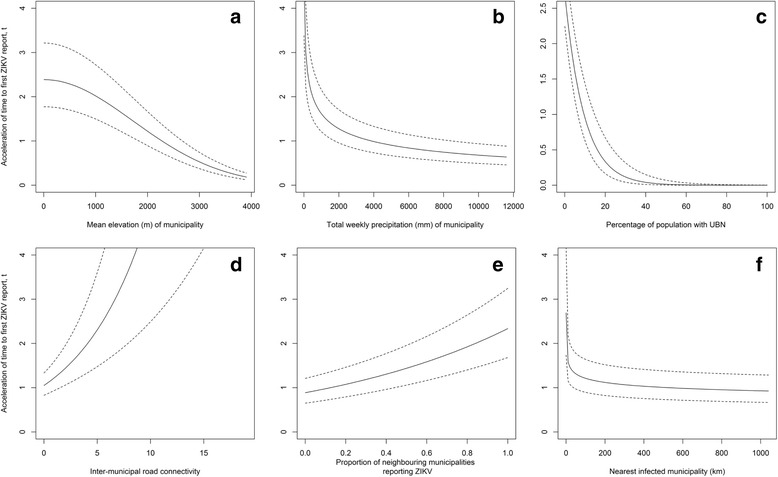
The accelerated failure times predicted by the best model during the period of October 24th 2015 to September 17th 2016 for the effects of municipality elevation (m) (**a**), total weekly precipitation (mm) (**b**), percentage of municipal population with unsatisfied basic needs (UBN) (**c**), inter-municipal connectivity (**d**), proportion of neighbouring municipalities reporting ZIKV (**e**), and nearest municipality reporting ZIKV (km) (**f**)

In the best AFT model (Table 4), the environmental variables, particularly elevation and precipitation, had a significant negative impact on the time to the first reported ZIKV case in municipalities. That is, increases in elevation and precipitation slowed the expected time by a factor of 1.18 (i.e. 1/acceleration factor when the acceleration factor is < 1) and 1.41, respectively (Fig. 5a, b). Social determinants of ZIKV transmission also significantly affected the time to the first report. Specifically, an increase in the percentage of the municipal population with UBN delayed the expected time by a factor of 1.07 (Fig. 5c), while inter-municipal connectivity accelerated the expected time by a factor 1.17 (Fig. 5d). The significant interaction of these variables indicated that more wealthy areas reported a first case more quickly, especially when inter-municipal connectivity was high (Additional file 3: Figure S3.2.b). As for neighbourhood disease intensity variables, an increase in proportion of infected neighbouring municipalities accelerated significantly the expected first reporting time by a factor of 2.63 (Fig. 5e). Neighbouring infected municipalities were associated with decreasing the time to the first report when located, on average, within about 100 km (Fig. 5f). Increasing distance to the nearest infected municipality slowed down the expected time of first report by a factor 1.12. See Additional file 3 for details on candidate models and best model variable effects defined by survival and hazard functions.

**Table 4:** Parameters of the best model estimating time to first report of ZIKV in municipality *i* at week *t*

Variable	Weight mean	Coef	SE(Coef)	Wald *P*	AF
Mean elevation	4.02	-0.168	0.008	< 0.01	0.85
Total weekly precipitation (term 1)	-2.55	-0.323	0.028	< 0.01	0.71
Total weekly precipitation (term 2)	13.60	-0.023	0.003	< 0.01	
UBN	5.98	-0.076	0.021	< 0.01	0.93
Connectivity	2.79	0.157	0.029	< 0.01	1.17
Proportion of neighbours reporting ZIKV	0.252	0.968	0.093	< 0.01	2.63
Distance to nearest municipality reporting ZIKV	-1.30	-0.115	0.025	< 0.01	0.89
UBN X connectivity		-0.014	0.004	< 0.01	0.99
Baseline parameters					
log(scale)		3.274	0.154	< 0.01	
log(shape)		0.524	0.025	< 0.01	

*Notes*: *AF* acceleration factor, *UBN* unsatisfied basic needs, *SE* standard error. See Additional file 3 for definitions of variable functional form transformations

## Discussion

Public health management of ZIKV is informed by the timely identification of vulnerable populations. In this study we used two modelling approaches to characterize population vulnerability as indicators for sustained autochthonous ZIKV transmission and timing to emergence. Results from our dual-modelling approach provide insights as to the roles of environmental, social and neighbourhood disease intensity factors on ZIKV emergence patterns.

The probability of reporting ZIKV was higher at warm temperatures and the timing to its detection was faster at lower elevations. Elevation is correlated with temperature (lower elevations being warmer) but may also be a proxy for different habitats that have more complex interactions with mosquito survival and reproduction [15]. Warmer regions support larger vector populations *via* shorter vector generation times, and higher survival rates as well as shorter virus generation times resulting in higher rates of infective bites [13, 14, 44]. Nighttime temperature was a better predictor for ZIKV emergence than daytime temperature. In Colombia, nighttime temperatures drop below the suitable range for *Aedes* species reproduction and survival of 16–35 °C more often than daytime temperatures exceed this range [13]. As elevation increases, so too does the range in daily temperature, and this has been found to negatively impact *Ae. aegypti* survival and potential to transmit dengue [15, 45]. The estimated survival and hazard functions, given the range of elevation in Colombia, were in close agreement with a study reporting *Ae. aegypti* to be abundant up to 2200 m in Colombia (Additional file 3: Figure S3.2.c) [46].

Higher total precipitation over the study period was associated with a decrease in the probability of reporting ZIKV. Also, higher total weekly precipitation in the current week delayed detection. Higher rainfall is generally linked with larger mosquito populations and disease transmission; however, the effects of rainfall are exerted through complex pathways [47, 48]. For example, in a laboratory setting lower precipitation reduced larval survival more acutely at higher temperatures for *Ae. albopictus* [44]. The relationship between rainfall and *Aedes* spp*.* abundance can be obscured if containers providing larval habitat are covered and managed by people, thus, independent of rainfall [49, 50]. Decreased rainfall can lead to people filling storage containers and providing more mosquito breeding habitat [50, 51] while heavy rainfall can flush and destroy breeding habitat [52]. We would expect many of these factors to be detected as time-lagged effects for precipitation, given their potential to influence future mosquito population abundance [20]. The main effect of precipitation (when assessed on a weekly time-scale) was essentially an immediate rather than a time-lagged effect. Therefore, we may be detecting an effect of rainfall on human behaviour, in that, ill people are less likely to travel to health centres when it is raining. This is the most likely explanation as the observed association was between precipitation during the week of reporting which would not have had time to impact ZIKV transmission via effects on mosquito reproduction or activity.

The role of poverty on vector-borne disease dynamics is also complex [53]. Impoverished areas have been linked to higher disease incidence [16, 18]. Poor quality services, such as insufficient plumbing, lead to water storage containers and more standing water providing greater breeding habitat and consequently higher vector abundance [17]. Further, poor quality housing, such as broken or absent screens on windows, can increase human exposure [54]. However, we observed a negative relationship between poverty level and detection of ZIKV, and time to the first reported ZIKV case. It may be that poor areas have more limited access to health centres, and thus fewer people are reporting suspect cases for investigation [19]. Inter-municipal connectivity can also be a proxy for infected people spreading infection (i.e. small-scale human movements) [55]. In our statistical models we found evidence that lower inter-municipal connectivity in poor areas reduces the detection of ZIKV, and increases the time to the first reported case given the significant interaction between the connectivity and poverty variables. Poorer areas may also have lower access to education highlighting the presence of the epidemic, and the need to report suspect cases [20]. We do not suggest that poorer areas are less vulnerable to sustain autochthonous transmission than wealthy areas, though further studies are needed to determine the mechanism(s) behind the negative association we detected.

Through our dual-modelling approach we detected different aspects of the emergence dynamics as reflected through the temporal scales of the environmental variables retained in the best models. The logistic regression approach detected the influence of temperature and precipitation over longer-term (study period mean) rather than shorter-term (weekly and lagged means) time scales. Using mean environmental data from across the study period is likely better at defining areas of habitat suitability and vector occurrence than short time-scale trends, as also found for an index of *Aedes* spp. vector habitat and arbovirus transmission suitability [31]. Even if weekly temperature and precipitation are suitable for vectors over short periods, conditions likely need to be sustained year-round for vectors to survive. Conversely, the AFT best model retained the shorter-term total weekly precipitation variable rather than total study period precipitation, although, as described above, the effect of precipitation was likely on human behaviour. The survival model also provides a reference value for the distance (within 100 km) over which infected municipalities may most likely influence the timing of ZIKV emergence. This result underlines the importance of having effective surveillance systems that can use detection of disease in one location to act as early warning indicators for nearby populations.

Maps of estimated population vulnerability in countries newly affected by ZIKV can guide surveillance and target prevention and preparedness strategies. Using the logistic regression model we estimated the population vulnerability to sustained autochthonous transmission of ZIKV given our long-term temperature and precipitation covariates, and road connectivity. We did not include effects of poverty because we think its effect was mostly driven by underreporting rather than impacts on transmission. Also we did not account for the spatial progression of ZIKV, though this can be useful for management preparedness if the goal is to estimate population vulnerability relative to locations of index cases or areas at high risk to disease importation. Whether our model can be generalised to other tropical and sub-tropical countries remains to be studied. However if it can, our approach may have the ability to predict vulnerability of ZIKV infection at a local scale more widely in Central and South America.

Study limitations arise from the quality and availability of surveillance data. The spatial progression of ZIKV through Colombian municipalities is mostly clustered but there are notable outliers. Municipalities in which ZIKV was not detected could be the result of (i) inadequate surveillance (false negatives from asymptomatic or sub-clinical infections, underreporting when clinical infections were missed, misdiagnosis with other diseases) [2, 56, 57]; (ii) virus absence where ZIKV transmission is possible (true negatives); or (iii) virus absence where ZIKV transmission is not possible, such as in areas of high elevation with sub-zero temperatures (true negatives). To reduce error from inadequate surveillance we grouped suspected and confirmed ZIKV cases. Suspected case data have been used to reliably represent ZIKV dynamics [58]. Yet, we may have overestimated the effect of the variables retained in our models if suspected cases were misdiagnosed from co-circulating dengue and chikungunya [56, 57]. We expect this error to be low given that INS diagnostic protocols tested for these diseases if they were known to be circulating in areas where the patient lived or had visited.

We were also limited by not having surveillance data reported prior to January 9th, 2016. Surveillance data are available from the Pan American Health Organization at the website http://www.paho.org/hq/index.php?options=com_content&view=article&id=12390&Itemid=42090&lang=en), but exist at the country level. We backtracked case counts retrospectively for municipalities with counts greater than 1 on January 9th, 2016 assuming cases doubled weekly [36]. Our assumption may fail in areas where transmission dynamics do not follow this exponential growth rate, as could be caused by very high or low rates of movement of infected people [55]. Even if we had available surveillance data prior to 2016, these earlier data had higher rates of underreporting from areas where ZIKV circulation had yet to be confirmed and suffered from changing diagnostic protocols [59, 60]. Also, under-reporting is commonplace in disease surveillance when an estimated 80% of cases are asymptomatic [2]. The first reported case in a municipality is unlikely to be the first actual case given the true number of infections. However, the difference in timing for the actual first case occurring in Colombia and the detection of the first cases by surveillance is expected to be longer, than the time lag between a first case in a municipality and detection of cases by surveillance after the ZIKV outbreak had been recognised and surveillance systems alerted.

All of these issues could affect the coefficients of variables in the final models. However, it would be expected that these issues would affect all locations equally (with inter-municipality variations in reporting rates being largely accounted for by the poverty metric), and while the precise coefficient values would be different if all cases were captured, the variables identified as significant, and their relative importance as determinants of local vulnerability, should be robust.

## Conclusions

Public health management of ZIKV has been challenged by the rapid spread of this disease through the Americas. We present a quantitative approach using two modelling frameworks that are relatively easy to implement and interpret. The logistic regression and AFT models focused on different aspects of the emergence dynamics. The logistic regression model detected variables associated with environmental suitability for vector abundance and ZIKV transmission, while the survival model detected finer-scale processes affecting the timing of emergence. We suggest effective management of ongoing and future ZIKV infection will benefit from being able to both geo-locate areas of at risk for sustained autochthonous transmission, and predict the timing of emergence in the vulnerable areas. Therefore, limited public health resources can be focused more accurately on high risk locations and times.

## Additional files

Additional file 1: Case definitions for Zika virus infection in Colombia. 1.1 Suspected cases. 1.2 Confirmed cases. 1.3 References. (DOCX 31 kb)

Additional file 2: Methodology. **2.1** Data cleaning. **Table S2.1.a.** Number of observations and percentage of missing data. **Table S2.1.b**. Model parameter estimates, standard error and *t*-value. **Table S2.1.c**. Model parameter estimates, standard error and *t*-value. **Table S2.1.d**. Correlation between existing and imputed data. **2.2** Explanatory variables. **2.2.1** Environmental. **2.2.2** Social. **2.2.3** Neighbourhood disease intensity. **Table S2.2.3.a.** Summary statistics for explanatory variables. **2.3** Model formulation for a binary longitudinal response variable. **2.3.1** Logistic regression. **2.3.2** Accelerated failure time model. **2.4** References. (DOCX 56 kb)

Additional file 3:Model Output. **3.1** Logistic regression **Table S3.1.a.** Parameter estimates, standard errors, t-values and p-values. **Table S3.1.b.** Variable functional forms. **Figure S3.1.a.** Probability of reporting a first case given a) mean study period nighttime temperature (°C), b) total study period precipitation (mm), c) UBN at high and low levels of inter-municipal road connectivity, and d) proportion of neighbouring municipalities reporting ZIKV. **3.2** Accelerated failure time model. **Figure S3.2.a.** a) Density, b) survivor and c) hazard functions at mean covariate values. **Table S3.2.a.** Variable functional forms. **Figure S3.2.b.** Acceleration time to a first case given UBN and inter-municipal connectivity, and other model variables at their median values. **Figure S3.2.c.** (i) survival and (ii) hazard functions given a) municipality elevation (m), b) total weekly precipitation (mm), c) UBN, d) inter-municipal connectivity, f) proportion of neighbouring municipalities reporting ZIKV, and g) nearest municipality reporting ZIKV (km). **3.3** Variable effects. **3.3.1** AIC model comparisons. **Table S3.3.1.a.** ∆AIC between best and candidate models. **Table S3.3.1.b.** ∆AIC between best and candidate models. **3.4** Model validation. **Table S3.4.a** 10-fold cross-validation folds for training and validation data and their mean predicted $t={e}^{x_i^{\prime}\beta }\ \tau, \kern0.5em t>0$ and difference (i.e. error) between their values. **3.5** References. (DOCX 1161 kb)

## Abbreviations

AF: Acceleration factor
AFT: Accelerated failure time
AIC: Akaike’s information criterion
AUC: Area under the curve
DANE: Departamento Administrativo Nacional de Estadística
EW: Epidemiological week
PAHO: Pan American Health Organization
ROC: Receiver operating characteristic
UBN: Unsatisfied basic needs
ZIKV: Zika virus
