# Supplementary material for: Environmental and social determinants of population vulnerability to Zika virus emergence at the local scale
Source: Parasit Vectors. 2018 May 8;11:290. doi: 10.1186/s13071-018-2867-8 (PMC5941591; doi:10.1186/s13071-018-2867-8)
Supplement: Supplementary file 1 — Case definitions for Zika virus infection in Colombia. 1.1 Suspected cases. 1.2 Confirmed cases. 1.3 References. (DOCX 31 kb) [file 13071_2018_2867_MOESM1_ESM.docx]

Additional File 1: Case definitions for Zika virus infection in Colombia

Erin E. Rees, Tatiana Petukhova, Mariola Mascarenhas, Yann Pelcat and Nicholas H. Ogden

Zika virus (ZIKV) case definitions are defined in a Colombian Government report [1] available at: <http://bvs.minsa.gob.pe/local/MINSA/3449.pdf>, as accessed on June 11^th^, 2017. There are a total of 1179 municipalities and sub-municipalities listed in the surveillance reports. We focused on municipalities (n=1065) and removed three island municipalities to concentrate on mainland disease spread. For an overview of the Colombian surveillance system interested readers can refer to Pacheco et al [2].

**AF1.1 SUSPECTED CASES**

**At-risk population**: neonates, children less than 1 year old, pregnant women, people aged 65 years and over with / without comorbidities

Clinical symptoms include exanthema and elevation of axillary body temperature greater than 37.2^0^C with one or more of the following symptoms that are not explained by other medical conditions: non-purulent conjunctivitis or conjunctival hyperemia, rash, arthralgia, myalgia, headache or general malaise. Additionally, the individual has visited places less than 2,200 m in Colombia and/or countries with or without confirmed circulation of ZIKV.

Blood or cerebral spinal fluid (CSF) samples were taken at the time limits established for laboratory confirmation of ZIKV infection.

**General population from abroad**, with or without confirmed circulation of the virus in their country of origin

Clinical symptoms include exanthema and elevation of axillary body temperature greater than 37.2^0^C with one or more of the following symptoms that are not explained by other medical conditions: non-purulent conjunctivitis or conjunctival hyperemia, pruritus or rash, arthralgia, myalgia, headache or general malaise. Additionally, the individual has come from or visited places less than 2,200 m in Colombia and/or countries with or without confirmed circulation of ZIKV. Blood or CSF samples were taken at the time limits established for laboratory confirmation of ZIKV infection without exception.

**General population coming from Colombian municipalities,** without confirmed ZIKV transmission

Clinical symptoms include exanthema and elevation of axillary body temperature greater than 37.2 ^0^C with one or more of the following symptoms that are not explained by other medical conditions: non-purulent conjunctivitis or conjunctival hyperemia, pruritus or rash, arthralgia, myalgia, headache or general malaise. Additionally, the individual has come from or visited places less than 2,200 m in Colombia and/or countries with or without confirmed circulation of ZIKV. Blood or CSF samples were taken for diagnostic confirmation according to the sampling protocol of areas without confirmation of ZIKV circulation, therefore, could also be tested for other viruses such as Dengue or Chikungunya.

**AF1.2 CONFIRMED CASES**

**Municipalities with confirmed ZIKV transmission for general population and at-risk populations** (neonates, neonates, children less than 1 year old, pregnant women, people aged 65 years and over with/without comorbidities)

Clinical symptoms include symptoms include rash, elevation of axillary body temperature greater than 37.2 ^0^C with one or more of the following symptoms that are not explained by other medical conditions: non-purulent conjunctivitis or conjunctival hyperemia, pruritis or rash, arthralgia, myalgia, headache or general discomfort. Additionally, the individual has been in sites less than 2,200 m in Colombia where there was confirmed autologous circulation of ZIKV 15 days prior to symptom onset.

**Laboratory confirmed cases**

Laboratory confirmed cases have a suspected case definition and a positive result for ZIKV by RT-PCR ZIKV or serology. Laboratory analyses were conducted at the National Reference Laboratory of Virology, from the National Network of Laboratories, National Institute of Health, or collaborating centres designated by the INS.

**AF1.3 REFERENCES**

1. Tolosa Pérez N, Ospina Martinez ML, Martinez Duran ME, Pacheco Garcia OE, Quijada Bonilla H. Protocolo de Vigilancia en Salud Pública: Enfermedad por Virus Zika. Inst Nac Salud. 2016;Versión 01: 1–27.

2. Pacheco O, Beltrán M, Nelson C a, Valencia D, Tolosa N, Farr SL, et al. Zika Virus Disease in Colombia - Preliminary Report. N Engl J Med. 2016; 1–10. doi:10.1056/NEJMoa1604037
