# Supplementary material for: Environmental and social determinants of population vulnerability to Zika virus emergence at the local scale
Source: Parasit Vectors. 2018 May 8;11:290. doi: 10.1186/s13071-018-2867-8 (PMC5941591; doi:10.1186/s13071-018-2867-8)
Supplement: Supplementary file 2 — Methodology. 2.1 Data cleaning. Table S2.1.a. Number of observations and percentage of missing data. Table S2.1.b. Model parameter estimates, standard error and t-value. Table S2.1.c. Model parameter estimates, standard error and t-value. Table S2.1.d. Correlation between existing and imputed data. 2.2 Explanatory variables. 2.2.1 Environmental. 2.2.2 Social. 2.2.3 Neighbourhood disease intensity. Table S2.2.3.a. Summary statistics for explanatory variables. 2.3 Model formulation for a binary longitudinal response variable. 2.3.1 Logistic regression. 2.3.2 Accelerated failure time model. 2.4 References. (DOCX 56 kb) [file 13071_2018_2867_MOESM2_ESM.docx]

Additional File 2: Methodology

Erin E. Rees, Tatiana Petukhova, Mariola Mascarenhas, Yann Pelcat and Nicholas H. Ogden

**AF2.1 DATA CLEANING**

There were missing data in the time series of surface daytime and nighttime temperature data existing as 8-day averages from Moderate Resolution Imaging Spectroradiometer (MODIS) [1]. First we converted the data into weekly values, in that the calendar week containing at least four days of the 8-day average would be assigned the value of the 8-day average. Once in a weekly format we found that there was about 10% missing data (Table 2.1.a).

**Table** **2.1.a** Number of observations and percentage of missing data.

| Variable | Sample size | Missing data (%) |
| --- | --- | --- |
| nighttime temperature at week *t* | 45038 | 10.81 |
| daytime temperature at week *t* | 45912 | 9.08 |

We imputed missing data using a two-step approach. In the first step, data were imputed using a predictor variable. The nighttime and daytime data are highly correlated (at week t: r = 0.77, p-value <0.01). We used a mixed-effects model to estimate nighttime temperature given daytime temperature. The model was specified as a random intercept model using the municipality as a random effect, given that the time series of observations with municipalities were not independent of each other (Table 2.1.b).

**Table 2.1.b** Model parameter estimates, standard error and t-value.

| Parameters | β | Std. Error | t-value |
| --- | --- | --- | --- |
| Intercept | 8.64 | 0.200 | 43.26 |
| Daytime temperature at week t | 0.25 | 0.004 | 57.64 |
| Municipal deviation | 2.01 | 0.138 |  |

The nighttime temperature variable, not including the newly imputed data, was then used as a predictor for imputing the daytime temperature data using a mixed-effects model with municipality as a random effect (Table 2.1.c).

**Table 2.1.c** Model parameter estimates, standard error and t-value.

|  | β | Std. Error | *t*-value |
| --- | --- | --- | --- |
| Intercept | 20.91 | 0.124 | 168.23 |
| Daytime temperature at week t | 0.315 | 0.005 | 64.77 |
| Municipal deviation | 1.12 | 0.05 |  |

To assess the validity of the models to impute missing data, we calculated the correlation between the predicted data and the existing data. These data were highly correlated, thus, provided evidence that our imputation approach was appropriate (Table 2M2.1.d).

**Table** **2.1.d** Correlation between existing and imputed data.

| Variable | Correlation | *p*-value |
| --- | --- | --- |
| nighttime temperature at week *t* | 0.927 | < 0.01 |
| daytime temperature at week *t* | 0.867 | < 0.01 |

After imputing the data in this first step, there remained 4.8% missing data for both the nighttime and daytime temperature variables. We used a second step to further impute the data, under the assumption that observations closer in time are more likely to be similar in temperature than when further apart in time, over the short-term. To impute, we averaged existing observations together to estimate a missing value. For instance, if temperature of week *t* was unknown, we average the temperatures at weeks *t*-1 and *t*+2 to produce an estimate for time *t*. If temperature at weeks *t* and *t*+1 were unknown, we used the mean of weeks *t*-2 and *t*-1 to estimate for *t*, and the mean of weeks *t*+2 and *t*+3 to estimate for *t*+1. We did not have cases of three consecutive unknown values.

**AF2.2 EXPLANATORY VARIABLES**

**AF2.2.1 Environmental**

Vector environmental suitability is a previously developed composite measure of *Aedes* *species* vector habitat and arbovirus transmission suitability [2]. This variable is a function of i) temperature suitability for dengue transmission to humans via *Ae. aegypti*, ii) temperature suitability for dengue transmission to humans via *Ae. albopictus*, iii) minimum relative humidity, iv) annual cumulative precipitation, v) an enhanced vegetation index, and vi) urban versus rural habitat type (for details see [2]). The spatial resolution for the variable is at a 5 km grid resolution and the values were considered constant for our study period.

We used weekly total precipitation data (time-varying) at 0.1 degree latitude/longitude resolution from Integrated Multi-satellitE Retrievals for GPM (IMERG) from NASA [3]. We also created a variable from these data for the total precipitation for the study period (time-fixed) for each municipality.

Surface daytime and nighttime temperature were acquired as data as 8-day averages were acquired with 0.05 degree latitude/longitude resolution from Moderate Resolution Imaging Spectroradiometer (MODIS) [1] and translated into mean weekly (time-varying) daytime and nighttime temperature variables (Additional File 2). We also created variables for mean temperature over the study period temperatures from these data (i.e. time-fixed data). The mean weekly and mean study period variables for the precipitation and temperature data were included to assess effects on mosquito vectors and ZIKV transmission that may operate at different time scales. For example, a temperature variable with a one-week time lag may better capture the influence of EIP and recent vector activity and survival, whereas, the mean study period temperature may better capture environments suitable for long-term persistence of the mosquito vectors.

Elevation data were derived from a 90 m resolution digital elevation model [4].

**AF2.2.2 Social**

Data for municipal population size were available from the Colombian Government (DANE; *http://www.dane.gov.co*) and were converted to densities using municipality areas.

Poverty data came from unsatisfied basic needs (UBN) composite metric. UBN is a composite measure of five equally weighted metrics: i) inadequate dwellings, ii) crowding, iii) inadequate services, iv) economic dependence, and v) school-aged children not attending school [5]. Surveyed households must be classified by at least one metric to be considered as having UBN. UBN is a common measure of poverty for Latin America [6][5]. These data were available from the Departamento Administrativo Nacional de Estadística (DANE: *http://www.dane.gov.co*).

Municipal road density was considered a measure of human connectivity within a municipality, and obtained from the Center for International Earth Science Information Network (CIESIN) [7]. We used the same road network dataset to calculate a measure of inter-municipal connectivity as the number of roads entering/leaving the municipality.

**AF2.2.3 Neighbourhood disease intensity**

Neighbourhood disease intensity variables controlled for the reported spatial distribution and temporal progression of cases. The nearest infected municipality at time *t* was calculated as the Euclidean distance (km) from municipal centroid to centroid and represented the transmission distance. The proportion of infected neighbouring municipalities at *t* was determined from immediately adjacent municipalities and represented a spatial weight of transmission pressure. These variables were created using the cleaned and imputed surveillance data, in that, once a municipality had reported ZIKV it was assumed to remain a source of ZIKV over the course of the study period, even if cases were not reported in some weeks over the period. We consider this a valid assumption given that municipalities reporting ZIKV mostly showed an epidemiological curve typical of infectious disease incidence over time (i.e. increase, peak, decrease); furthermore, almost 99% of these municipalities continued reporting ZIKV by the last week in the study period. In the logistic regression, a categorical variable for reporting week accounted for the temporal progression of known ZIKV cases. This factor is a proxy for the growth rate of infection in the population.

Summary statistics for the explanatory variables used in building the logistic and AFT models are shown in Table 2.2.3.a. Variables are for municipalities reporting ZIKV at least once from 2015/10/24 to 2016/01/09. Results are shown for non-imputed data.

**Table 2.2.3.a.** Summary statistics for explanatory variables.

| **Variable** | **Mean** | **Median** | **Standard deviation** | **90% range** |
| --- | --- | --- | --- | --- |
| Mean study period temperature (°C) | 15.0 | 15.2 | 6.48 | 5.82-23.7 |
| Mean weekly daytime temperature (°C) | 25.5 | 25.6 | 5.74 | 18.1-32.7 |
| Mean weekly nighttime temperature (°C) | 14.8 | 15.3 | 7.01 | 5.25-23.8 |
| Total study period precipitation (mm) | 32590.2 | 28166.2 | 19615.5 | 11514.0-61344.6 |
| Total weekly precipitation (mm) | 622.9 | 263.3 | 861.4 | 1.56-1783.7 |
| Mean Elevation (m) | 1387.0 | 1387.1 | 991.1 | 77.7-2781.3 |
| Mean vector environmental suitability | 0.439 | 0.499 | 0.324 | 0.0-0.828 |
| Population density per km^2^ | 3.10 | 1.28 | 4.63 | 0.170-8.07 |
| Unsatisfied Basic Needs (% population) | 42.8 | 41.3 | 18.4 | 20.2-68.0 |
| Inter-municipal road connectivity | 3.39 | 3.0 | 2.82 | 0-7 |
| Road density per km^2^ | 0.096 | 0.088 | 0.069 | 0.00814-0.191 |
| Nearest infected municipality (km) | 41.7 | 17.2 | 67.3 | 8.95-111.11 |
| Proportion of neighbouring municipalities reporting ZIKV | 0.543 | 0.600 | 0.391 | 0.0-1.0 |

**AF2.3 MODEL FORMULATION FOR A BINARY LONGITUDINAL RESPONSE VARIABLE**

In this section we describe details of our modeling approaches by providing the theory of (i) a logistic regression model for a binary longitudinal response variable, and (ii) an accelerated failure time model for a binary longitudinal response variable.

**AF2.3.1 Logistic regression**

We modeled the probability of municipal *i* reporting at least 1 case of ZIKV in week *t* using a logistic regression model. Let $y_{it}\in\left\{ 0,1 \right\}$ be the binary response at week *t* (*t* = 1, …, *T*) from a municipality *i (i = 1, …, N)* such that *y_it_* = 1 if the *i*^th^ municipality reported at least 1 case of ZIKV at week *t*; otherwise, 0. Let *θ* denoted by $Y_{it}$be its generating random variable whose mean values are $P\left( Y_{it}=1 \right)=\theta_{it}$. The marginal probability of success is defined as:

$logit \theta_{it}=x_{it}^{'}\beta$, (1)

where $x_{it}$ is a *p*-dimensional vector of time-dependent explanatory variables with the corresponding regression coefficients $\beta$ and ($'$) represents the transpose. The dependence structure of the process is taken to be a second order of Markov chain that is parameterized such that the marginal parameter $\beta$ preserves its meaning regardless of the serial dependence. In a second order Markov chain, we consider the joint distribution of three components at the time ($Y_{i,t-2}, Y_{i,t-1}, Y_{it}$), where $t-2$ and $t-1$ are respectively two weeks and one week prior to time *t.* Imposing the constraints, the odds ratio of the joint distribution of a pair of binary responses is defined as:

$OR\left( Y_{i, t-1}, Y_{i,t-2} \right) = \psi_{1}= OR\left( Y_{i,t-1}, Y_{i,t} \right)$ (2)

$OR\left( Y_{i,t-2},Y_{i,t} | Y_{i,t-1}=0 \right)=\psi_{2}=OR\left( Y_{i,t-2},Y_{i,t} | Y_{i,t-1}=1 \right)$, (3)

where $\psi_{1}, \psi_{2}$ are two positive parameters. In this formulation, the serial dependence is regulated by $\lambda=\left( \lambda_{1}, \lambda_{2} \right)=(\log\psi_{1}, \log\psi_{2})$.

The logistic regression modeling process was performed using the R package bild ([*www.r-project.org*](http://www.r-project.org)). The serial dependence was assumed to be constant across time and municipalities. For more information, the reader is referred to [8][9].

This model structure enabled us to study risk of ZIKV emergence using temporally dynamic variables (e.g. proportion of neighbouring municipalities reporting ZIKV at week t), and still assess effects of temporally static variables (e.g. elevation, mean study period temperature) on the probability of reporting a first case of ZIKV at week t. Furthermore, we could also assess if the influence of temporally dynamic variables values (e.g. mean weekly temperature) for weeks leading up to a first reported case differed from the variable value at the week of the first reported case. If there was no difference, the variable would be a poor model predictor. If there was a difference and the variable values were similar for weeks preceding an outcome of *y_it_* = 0 and in areas never reporting cases during the study period, then the variable would be a good model predictor.

**AF2.3.2 Accelerated failure time model**

We also modeled the time to the first reported ZIKV case in municipalities via a survival regression model. To efficiently use all of the information we had about the observations, we adopted a parametric technique. In this approach, we specified the shape of the baseline hazard based on knowledge from reported research findings [10][11][12], among others. That is, assuming that the presence of the first reported ZIKV case accelerates the infection growth rate exponentially and then taking into consideration that the number of cases decays within a timeframe due to different processes [10], we characterized the time to the first report of ZIKV in municipalities with an accelerated failure time (AFT) model.

AFT models describe the relationship between the survival probabilities and explanatory variables and measure the direct effect of predictors on the survival times. This characteristic of AFT models facilitates the interpretation of the results because the parameters measure the effect of the corresponding explanatory variable on the mean survival time.

Let $\ln t$ be the natural $log$ of the time $t$ (in weeks) at which a municipality *i* either did not report the first ZIKV case ($y_{it}=0$) or reported the first case of ZIKV ($y_{it}=1$). The general form of an AFT model is then described as:

$\ln t= x_{i}^{'}\beta+\ln\tau or t=e^{x_{i}^{'}\beta} \tau, t>0$, (4)

where $x_{i}^{'}\beta$ is a linear combination of time-dependent explanatory variables with the corresponding regression coefficients $\beta$; ($'$) represents transpose; $\ln\tau$ is an error term on the natural $log$ scale with an appropriate distribution. From equation (4), it can be see that $\tau$ is the distribution of survival times when $x_{i}^{'}\beta=0$. Furthermore, equation (4) also indicates that the linear combination of predictors $x_{i}^{'}\beta$ in the model act additively on $\ln t$ or multiplicatively on $t$. That is, they accelerate or decelerate the passage of time by a multiplicative factor.

We can rearrange equation (4) as following:

$\tau=e^{-x_{i}^{'}\beta}t or \ln\tau=-x_{i}^{'}\beta\ln t$, (5)

where $e^{-x_{i}^{'}\beta}$ is known as the acceleration parameter.

AFT models generated by a baseline hazard $h_{0}$ is defined as:

$h\left( t; \theta\right)= g_{t}\left( t, \theta\right)h_{0}\left( g\left( t, \theta\right) \right),$ (6)

where $g$ is a positive function that helps define a parametric family of baseline survivor functions; $\theta$ is a parameter vector used in modeling the baseline distribution.

The AFT modeling process with a parametric baseline hazard was implemented on the survival ZIKV data using the aftreg function in the eha R package ([*www.r-project.org*](http://www.r-project.org)). We considered the Weibull and log-normal distributions of survival times based on knowledge of how failures arise [9][10][11] and insights into what we would be expected in terms of a hazard function. For the Weibull ZIKV survival times,$t\sim W\left( \lambda,p \right)$ where $\lambda>0$ is the scale parameter and $p>0$ is the shape parameter of the distribution, the error term $\tau$ should follow an extreme value distribution with the scale parameter $\lambda$ and the shape parameter $p=1/\sigma$ where $\sigma$ is the standard deviation [13]. For log-normal ZIKV survival times,$t\sim\ln N(\mu, \sigma)$, the error term$\tau$ should be normally distributed with the mean $\mu=0$ and the standard deviation $\sigma=1$ , $\tau\sim N(0,1)$ [13]. The model adequacy was assessed using diagnostic procedures based on Akaike’s information criterion (AIC) and from graphical examinations. The chosen model had the smallest AIC value with fewer parameters and satisfied the AFT properties. More information on AFT models for a binary longitudinal response variable can be found in [13][14].

**AF2.4 REFERENCES**

1. Z. Wan SH, Hulley G. MOD11C2 MODIS/Terra Land Surface Temperature/Emissivity 8-Day L3 Global 0.05Deg CMG V006. NASA EOSDIS Land Processes DAAC. https://doi.org/10.5067/MODIS/MOD11C2.006. 2015.

2. Messina JP, Kraemer MU, Brady OJ, Pigott DM, Shearer FM, Weiss DJ, et al. Mapping global environmental suitability for Zika virus. Elife [Internet]. 2016;5:e15272. Available from: https://elifesciences.org/content/5/e15272

3. Huffman GJ, Stocker EF, Bolvin DT, Nelkin EJ. last updated 2014: 3IMERGHH Data Sets. NASA/GSFC, Greenbelt, MD, USA, ftp://jsimpson.pps.eosdis.nasa.gov/NRTPUB/imerg/gis/2015/, Accessed 30/01/2016. 2014.

4. Jarvis A, Reuter HI, Nelson A, Guevara E. Hole-filled SRTM for the globe Version 4, available from the CGIAR-CSI SRTM 90m Database. 2008;

5. Bustamente Roldan J, Jaramillo Herrera C, Chamie M. Quality of life national survey methodology. Bogota, D.C., Colombia; 2011.

6. Angulo R, Diaz B, Pinzon R. Multidimensional Poverty in Colombia, 1997-2010. Essex. United Kingdom; 2013.

7. CIESIN. No Title. 2013.

8. Azzalini A. Logistic regression for autocorrelated data with application to repeated measures. Biometrika. 1994;81:767–75.

9. Goncalves MH, Cabral MS, Azzalini A. The R Package bild for the Analysis of Longitudinal Data. J. Stat. Softw. [Internet]. 2012;46:1–17. Available from: http://doi.wiley.com/10.1002/0471722073.ch7

10. Ogden NH, Fazil A, Safronetz D, Drebot M, Wallace J, Rees EE, et al. Risk of travel-related cases of Zika virus infection is predicted by transmission intensity. Parasit. Vectors [Internet]. 2017;10:1–9. Available from: http://dx.doi.org/10.1186/s13071-017-1977-z

11. Ferguson NM, Cucunuba ZM, Dorigatti I, Nedjati-Gilani GL, Donnelly CA, Basanez M-G, et al. Countering Zika in Latin America. Science (80-. ). 2015;353:353–4.

12. Nishiura H, Kinoshita R, Mizumoto K, Yasuda Y, Nah K. Transmission potential of Zika virus infection in the South Pacific. Int. J. Infect. Dis. [Internet]. International Society for Infectious Diseases; 2016;45:95–7. Available from: http://dx.doi.org/10.1016/j.ijid.2016.02.017

13. Hosmer DW, Lemeshow S, May S. Applied survival analysis: Regression modeling of time to event data, 2nd Edition. Hoboken, NJ.: Wiley; 2008.

14. Brostrom G. Event History Analysis with R. Boca Raton, Florida: CRC Press; 2012.

15. Galanova NS, Lemeshko BY, Chimitova E V. Using nonparametric goodness-of-fit tests to validate accelerated failure time models. Optoelectron. Instrum. Data Process. [Internet]. 2012;48:580–92. Available from: http://dx.doi.org/10.3103/S8756699012060064
