# Supplementary material for: Environmental and social determinants of population vulnerability to Zika virus emergence at the local scale
Source: Parasit Vectors. 2018 May 8;11:290. doi: 10.1186/s13071-018-2867-8 (PMC5941591; doi:10.1186/s13071-018-2867-8)
Supplement: Supplementary file 3 — Model Output. 3.1 Logistic regression Table S3.1.a. Parameter estimates, standard errors, t-values and p-values. Table S3.1.b. Variable functional forms. Figure S3.1.a. Probability of reporting a first case given a) mean study period nighttime temperature (°C), b) total study period precipitation (mm), c) UBN at high and low levels of inter-municipal road connectivity, and d) proportion of neighbouring municipalities reporting ZIKV. 3.2 Accelerated failure time model. Figure S3.2.a. a) Density, b) survivor and c) hazard functions at mean covariate values. Table S3.2.a. Variable functional forms. Figure S3.2.b. Acceleration time to a first case given UBN and inter-municipal connectivity, and other model variables at their median values. Figure S3.2.c. (i) survival and (ii) hazard functions given a) municipality elevation (m), b) total weekly precipitation (mm), c) UBN, d) inter-municipal connectivity, f) proportion of neighbouring municipalities reporting ZIKV, and g) nearest municipality reporting ZIKV (km). 3.3 Variable effects. 3.3.1 AIC model comparisons. Table S3.3.1.a. ∆AIC between best and candidate models. Table S3.3.1.b. ∆AIC between best and candidate models. 3.4 Model validation. Table S3.4.a 10-fold cross-validation folds for training and validation data and their mean predicted $t={e}^{x_i^{\prime}\beta }\ \tau, \kern0.5em t>0$t=exi′βτ,t>0 and difference (i.e. error) between their values. 3.5 References. (DOCX 1161 kb) [file 13071_2018_2867_MOESM3_ESM.docx]

Additional File 3: Model Output

Erin E. Rees, Tatiana Petukhova, Mariola Mascarenhas, Yann Pelcat and Nicholas H. Ogden

**AF3.1 LOGISTIC REGRESSION**

The best selected logistic regression model used explanatory variables mean study period nighttime temperature, total study period precipitation, the poverty metric for the percentage of population with unsatisfied basic needs (UBN), the metric for inter-municipal road connectivity, proportion of neighbouring municipalities reporting ZIKV at week t, and an interaction of UBN and connectivity, while accounting for study week (Table 3.1.a). The functional forms of variables used in the best model are defined in Table 3.1.b.

**Table 3.1.a.** Parameter estimates, standard errors, t-values and p-values.

| Variable | Estimate | Standard error | t-value | p-value |
| --- | --- | --- | --- | --- |
| Intercept | -12.00 | 0.97 | -12.40 | <0.01 |
| Mean study period temperature | 4.09 | 0.24 | 16.80 | <0.01 |
| Total study period precipitation | -0.55 | 0.09 | -5.98 | 0.04 |
| Unsatisfied basic needs (UBN) | -0.93 | 0.32 | -2.95 | <0.01 |
| inter-municipal road connectivity (Connect) | 0.95 | 0.25 | 3.86 | <0.01 |
| Proportion of neighbours reporting ZIKV | 0.14 | 0.04 | 3.80 | <0.01 |
| UBN x Connect | -0.37 | 0.16 | -2.38 | 0.02 |
| 2015/10/31 | 0.5594551 | 0.39229036 | -2.377 | 0.15 |
| 2015/11/07 | 1.0568166 | 0.48412331 | 1.426 | 0.03 |
| 2015/11/14 | 1.5465758 | 0.53111082 | 2.183 | <0.01 |
| 2015/11/21 | 2.0297307 | 0.55605601 | 2.912 | <0.01 |
| 2015/11/28 | 2.5373982 | 0.57325929 | 3.650 | <0.01 |
| 2015/12/05 | 3.1474222 | 0.58480602 | 4.426 | <0.01 |
| 2015/12/12 | 3.7327843 | 0.59040662 | 5.382 | <0.01 |
| 2015/12/19 | 4.2260924 | 0.59434338 | 6.322 | <0.01 |
| 2015/12/26 | 4.6611164 | 0.59745852 | 7.111 | <0.01 |
| 2016/01/02 | 5.0977657 | 0.59979440 | 7.802 | <0.01 |
| 2016/01/09 | 5.9028132 | 0.60113336 | 8.499 | <0.01 |
| 2016/01/16 | 6.1747191 | 0.60173912 | 9.819 | <0.01 |
| 2016/01/23 | 6.4647976 | 0.60156097 | 10.261 | <0.01 |
| 2016/01/30 | 6.7335026 | 0.60161493 | 10.747 | <0.01 |
| 2016/02/06 | 7.0157958 | 0.60255553 | 11.192 | <0.01 |
| 2016/02/13 | 7.1846737 | 0.60497950 | 11.643 | <0.01 |
| 2016/02/20 | 7.3173906 | 0.60736379 | 11.876 | <0.01 |
| 2016/02/27 | 7.4312032 | 0.61022693 | 12.048 | <0.01 |
| 2016/03/05 | 7.5167260 | 0.61406460 | 12.178 | <0.01 |
| 2016/03/12 | 7.5899337 | 0.61964834 | 12.241 | <0.01 |
| 2016/03/19 | 7.6349164 | 0.62533835 | 12.249 | <0.01 |
| 2016/03/26 | 7.6966738 | 0.63061919 | 12.209 | <0.01 |
| 2016/04/02 | 7.7523378 | 0.63689735 | 12.205 | <0.01 |
| 2016/04/09 | 7.7888394 | 0.64332441 | 12.172 | <0.01 |
| 2016/04/16 | 7.7961728 | 0.64738629 | 12.107 | <0.01 |
| 2016/04/23 | 7.8186262 | 0.65725549 | 12.043 | <0.01 |
| 2016/04/30 | 7.9063266 | 0.66190777 | 11.896 | <0.01 |
| 2016/05/07 | 7.9697312 | 0.66792255 | 11.945 | <0.01 |
| 2016/05/14 | 8.0065300 | 0.67608175 | 11.932 | <0.01 |
| 2016/05/21 | 8.0628510 | 0.68271332 | 11.843 | <0.01 |
| 2016/05/28 | 8.1387087 | 0.67563458 | 11.810 | <0.01 |
| 2016/06/04 | 8.1792622 | 0.68280167 | 12.046 | <0.01 |
| 2016/06/11 | 8.2385902 | 0.68909363 | 11.979 | <0.01 |
| 2016/06/18 | 8.2942872 | 0.69711255 | 11.956 | <0.01 |
| 2016/06/25 | 8.3108585 | 0.70459208 | 11.898 | <0.01 |
| 2016/07/02 | 8.3239403 | 0.71133495 | 11.795 | <0.01 |
| 2016/07/09 | 8.3552478 | 0.72055404 | 11.702 | <0.01 |
| 16/07/2016 | 8.3641878 | 0.72528487 | 11.596 | <0.01 |
| 23/07/2016 | 8.3892688 | 0.73447624 | 11.532 | <0.01 |
| 30/07/2016 | 8.4137958 | 0.74379656 | 11.422 | <0.01 |
| 06/08/2016 | 8.4145345 | 0.74285746 | 11.312 | <0.01 |
| 13/08/2016 | 8.4325001 | 0.75117143 | 11.327 | <0.01 |
| 20/08/2016 | 8.4477182 | 0.75840722 | 11.226 | <0.01 |
| 27/08/2016 | 8.4577538 | 0.76383385 | 11.139 | <0.01 |
| 03/09/2016 | 8.4697077 | 0.77092750 | 11.073 | <0.01 |
| 10/09/2016 | 8.4984181 | 0.78267645 | 10.986 | <0.01 |
| 17/09/2016 | 8.5041383 | 0.78715699 | 10.858 | <0.01 |
| log.psi1 | 0.2294357 | 0.22832527 | 10.804 | <0.01 |
| log.psi2 | 1.9095292 | 0.36368435 | 44.802 | <0.01 |

Parameters log.psi1 and log.psi2 control for serial dependence in the longitudinal binomial response variable using Markov chain with a second order of dependence

**Table 3.1.b.** Variable functional forms.

| Variable name, symbol | Functional form |
| --- | --- |
| Mean study period nighttime temperature (*X*_1_) | (*X*_1_ + 2.2)^0.5 |
| Total study period precipitation (*X*_2_) | log(*X*_2_ + 1.6) |
| UBN (*X*_3_) | (*X*_3_ + 2.1)^0.5 |
| Connectivity (*X*_4_) | *X*_4_ + 1.6 |
| Proportion of neighbouring municipalities reporting ZIKV (*X*_5_) | *X*_5_ + 1.5 |

The probability of reporting ZIKV was calculated at the week of peak reporting for municipal level index cases. Probabilities were calculated as a function of a chosen explanatory variable, while keeping the other model variables at their median value for the study period (Figure 3.1.a). For this we used raw rather than centred parameter values.

| (i)  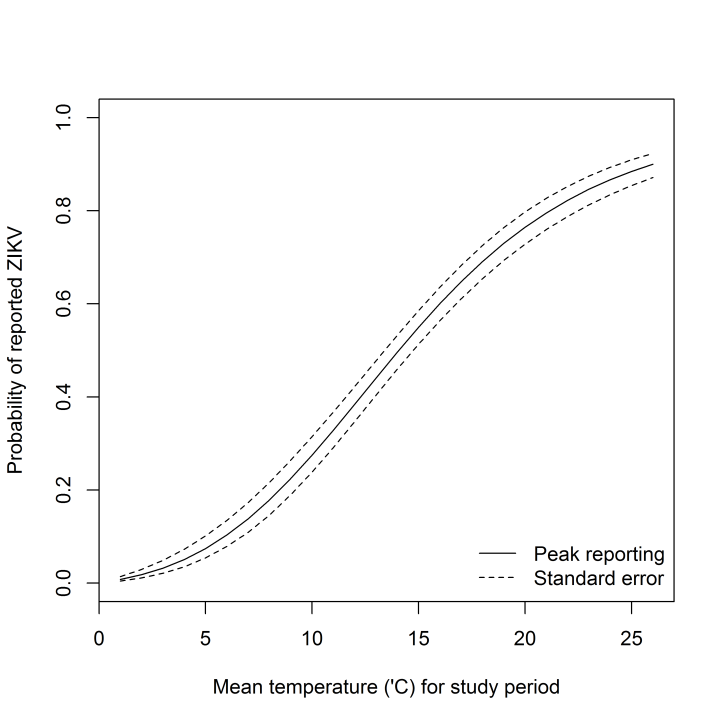 | (ii)  **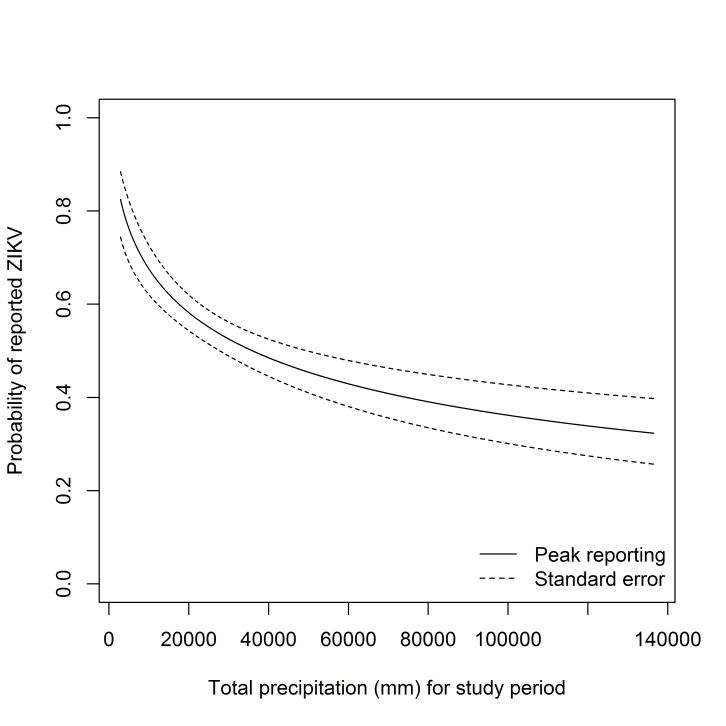** |
| --- | --- |
| (iii)  **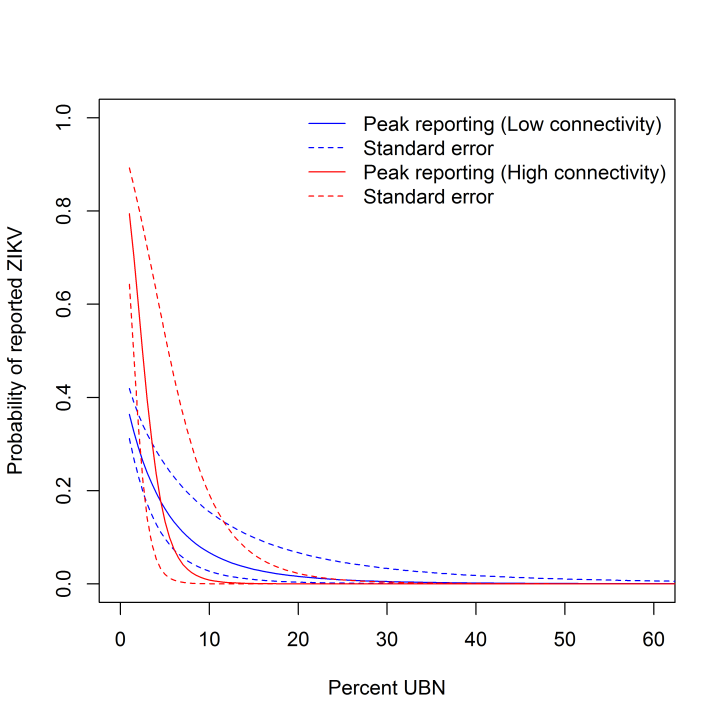** | (iv)  **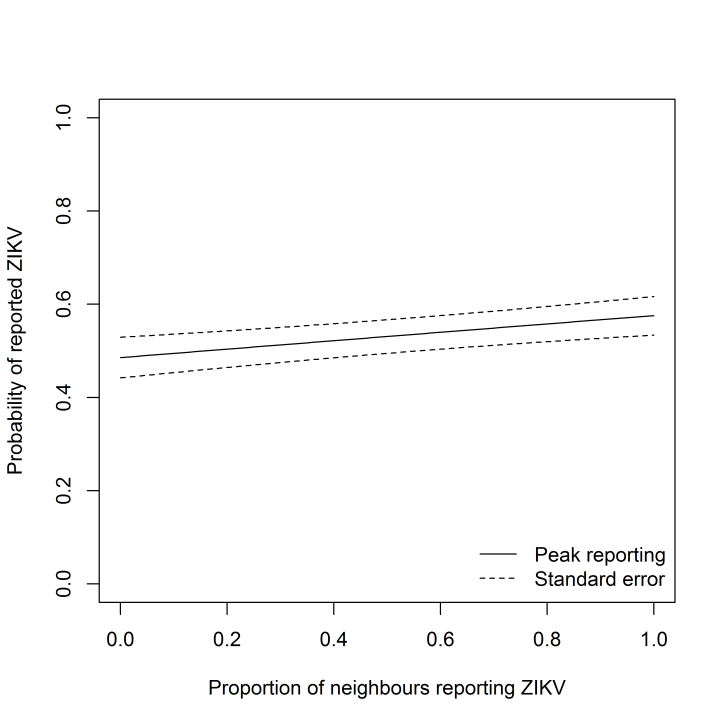** |

**Figure 3.1.a.** Probability of reporting a first case given a) mean study period nighttime temperature (°C), b) total study period precipitation (mm), c) UBN at high and low levels of inter-municipal road connectivity, and d) proportion of neighbouring municipalities reporting ZIKV.

**AF3.2 ACCELERATED FAILURE TIME MODEL**

The best model contained transformed mean municipal elevation, transformed weekly mean precipitation, the poverty metric UBN, inter-municipal connectivity, proportion of infected neighbouring municipalities, a log-transformed proximity of the municipality under consideration to the nearest infected municipality, and an interaction between UBN and connectivity (Figure 3.2.a). The functional forms of variables used in the top model are defined in Table 3.2.a. The interaction between UBN and inter-municipal connectivity indicated that wealthy areas reported a first case more quickly, especially when inter-municipal connectivity was high (Figure 3.2.b).

| (i)  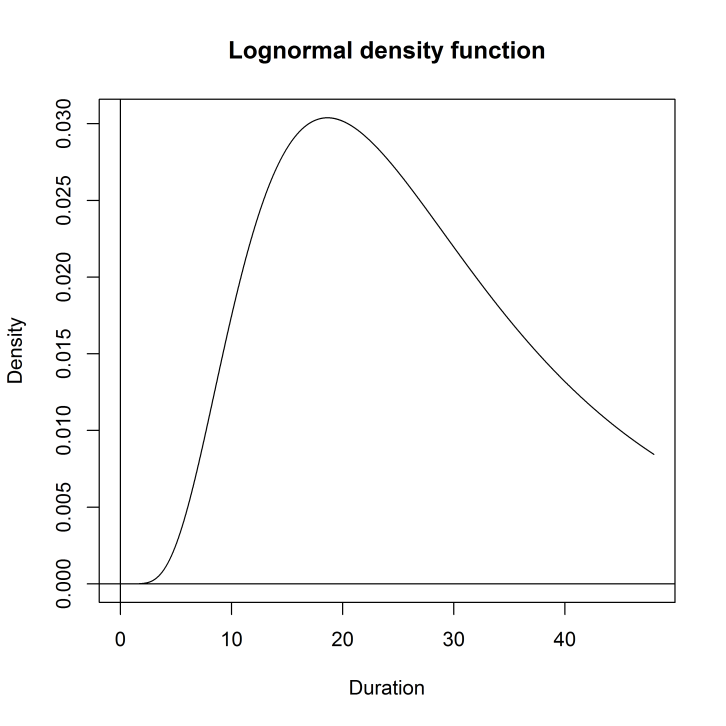 | (ii)  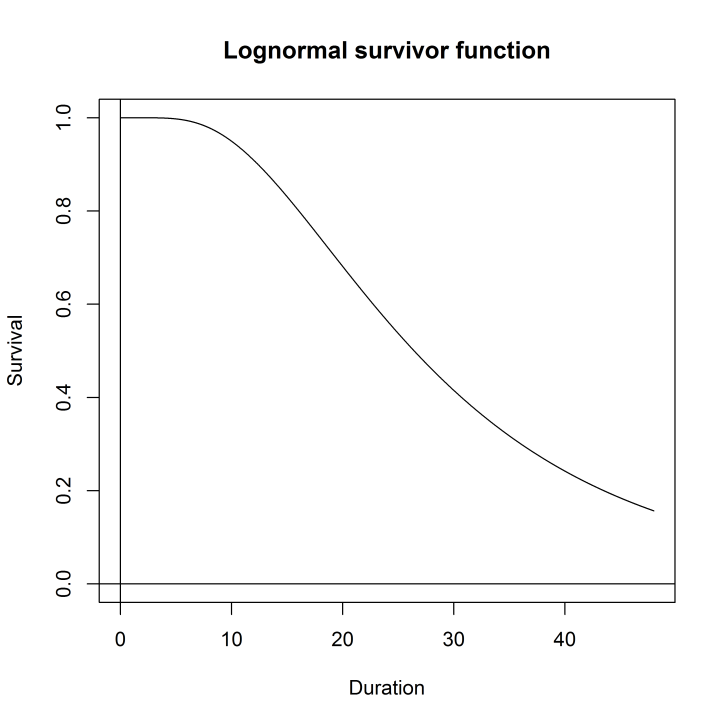 |
| --- | --- |
| (iii)  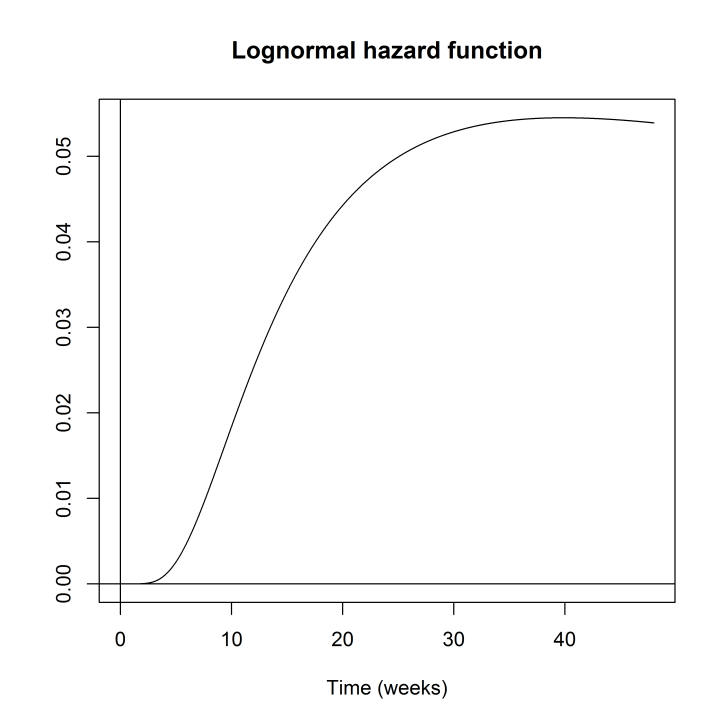 |  |

**Figure 3.2.a.** a) Density, b) survivor and c) hazard functions at mean covariate values.

**Table 3.2.a.** Variable functional forms.

| Variable name, symbol | Functional form |
| --- | --- |
| Mean elevation, *X*_1_ | (*X*_1_*+0.1)/1000)^2^ |
| Total weekly precipitation _t_, *X*_2_ | log((X_2_ + 0.1)/1000) |
| Total weekly precipitation _t-1_, *X*_3_ | log((X_3_ + 0.1)/1000) |
| Total weekly precipitation _t-2_, *X*_4_ | log((X_4_ + 0.1)/1000) |
| Total study period precipitation, *X_5_* | *X_5_*^0.5^/100 |
| Population density, *X*_6_ | log(*X*_6_+0.01) |
| Distance to nearest municipality reporting ZIKV, *X*_7_ | log((*X*_7_+0.1)/100) |

* Covariates were rescaled to have meaningful baseline hazards by subtracting the minimum value from each observation

| **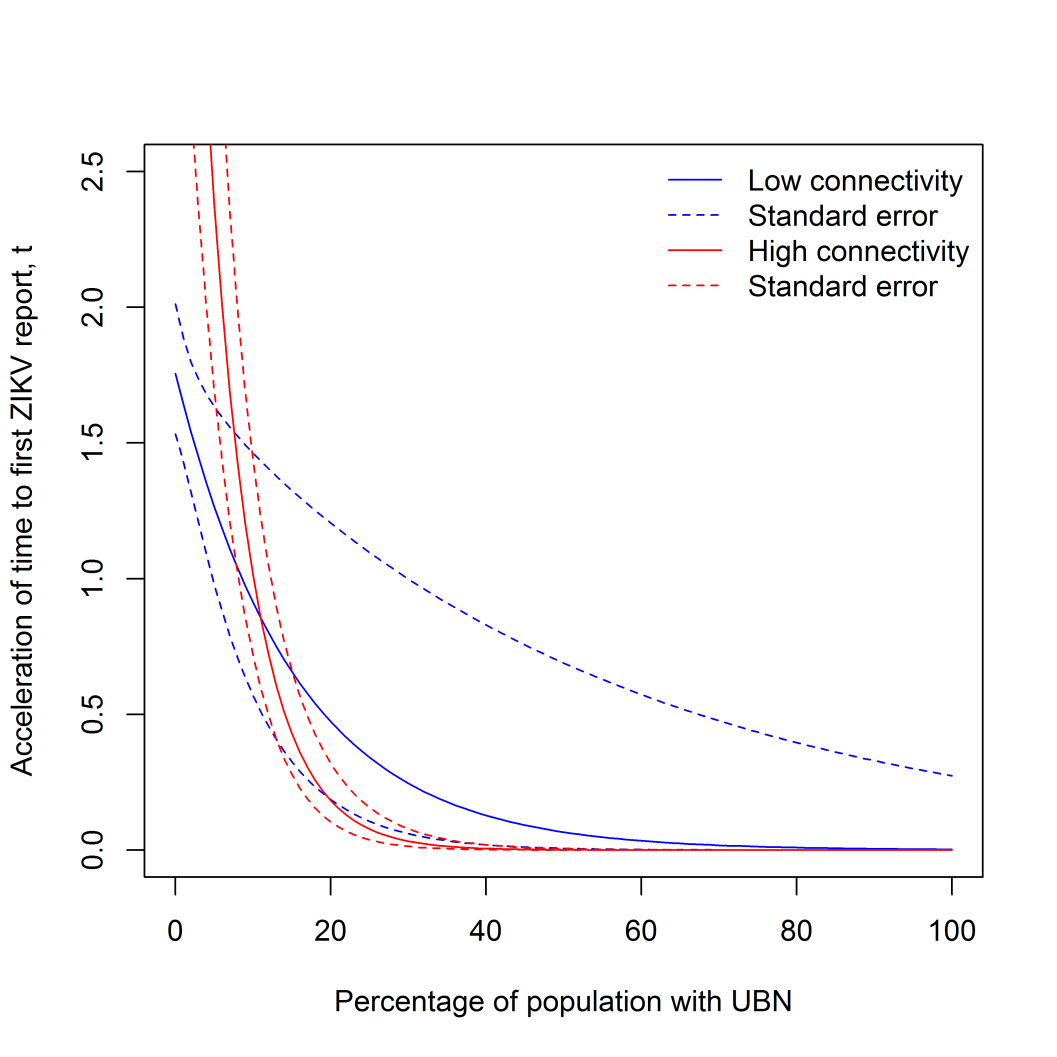** |
| --- |

**Figure 3.2.b.** Acceleration time to a first case given UBN and inter-municipal connectivity, and other model variables at their median values.

To help interpret the effects of variables retained in the AFT model on the timing of a first case of ZIKV being reported we plotted the probabilities of surviving and not surviving (hazard) over the range of variable values, while holding constant the other variables at their median value for the study period (except for the proportion of neighbouring municipalities reporting ZIKV, which we held constant at 0.5). We use the survival function to understand the probability of the event occurring at time *t*, and the hazard function to define the rate of events occurring at *t* (Figure 3.2.c).

| A(i)  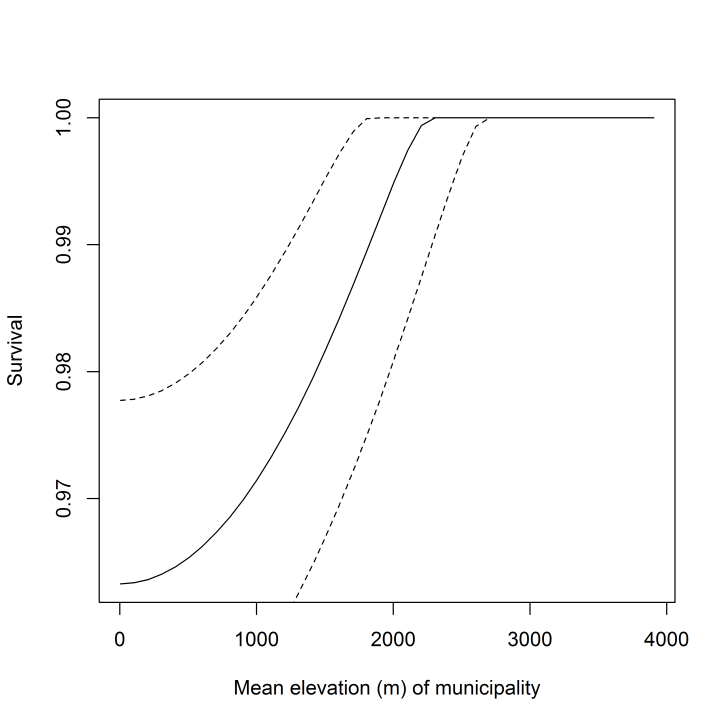 | (ii)  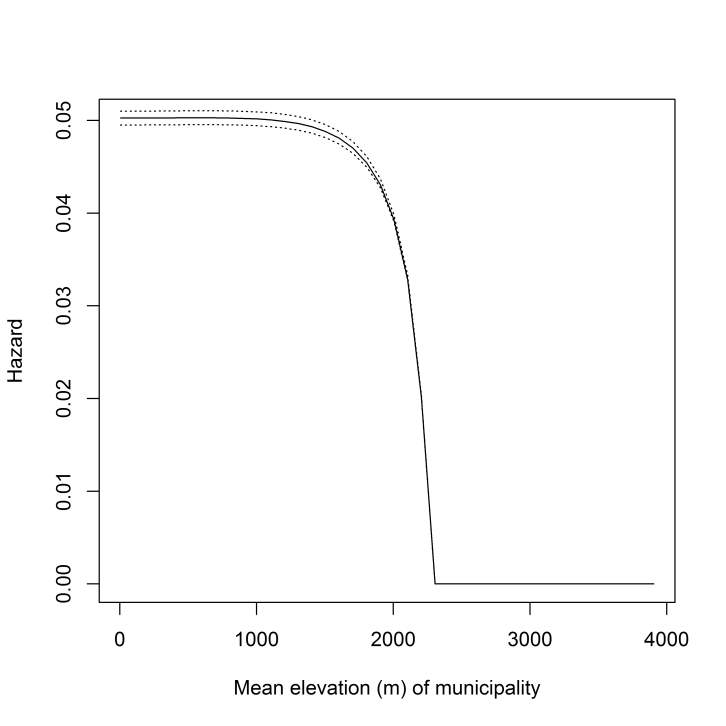 |
| --- | --- |

| B(i)  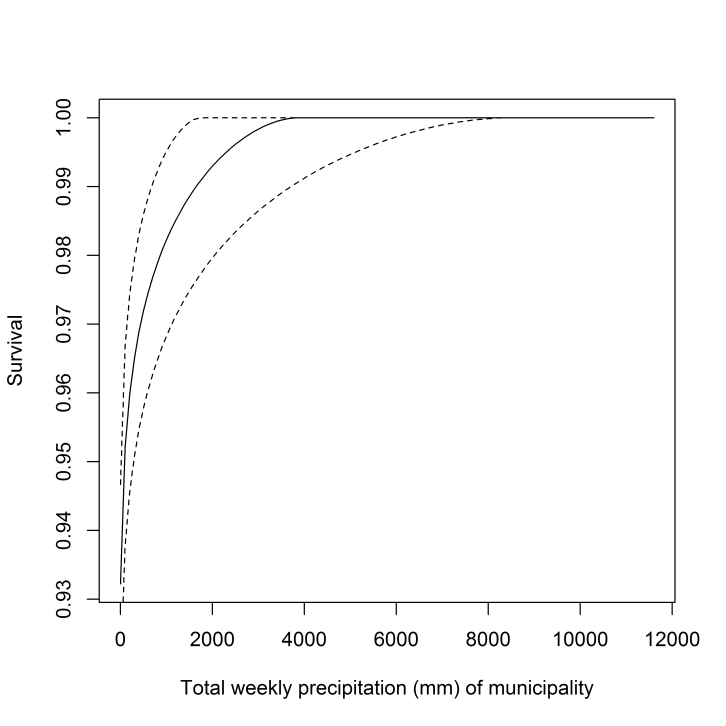 | (ii)  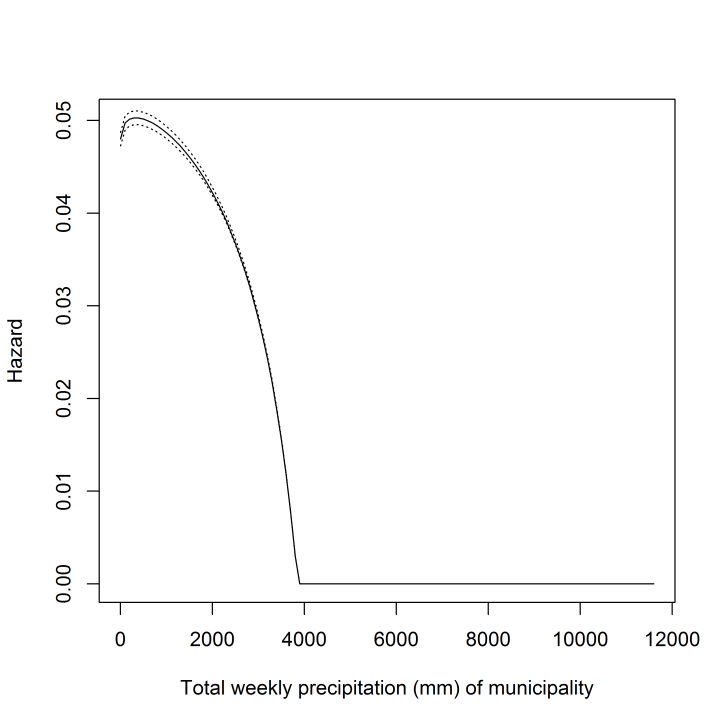 |
| --- | --- |

| C(i)  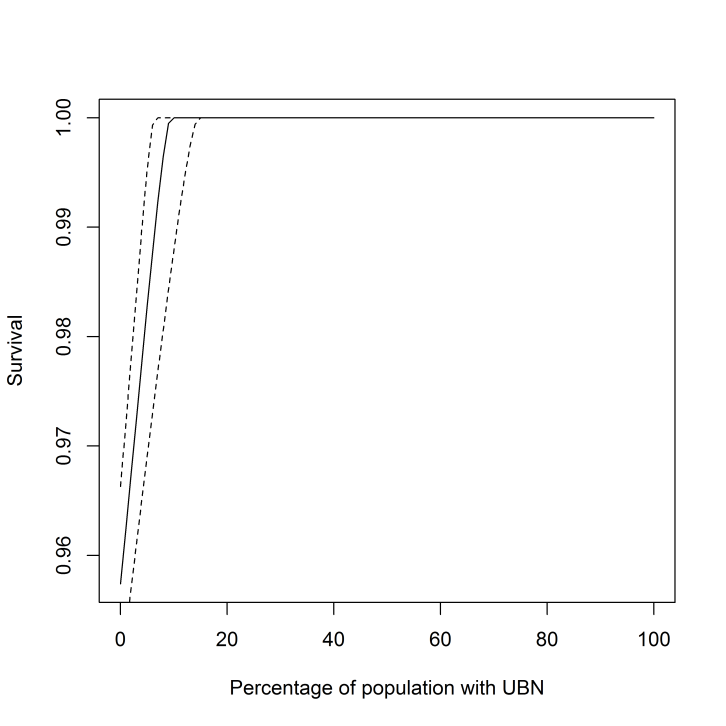 | (ii)  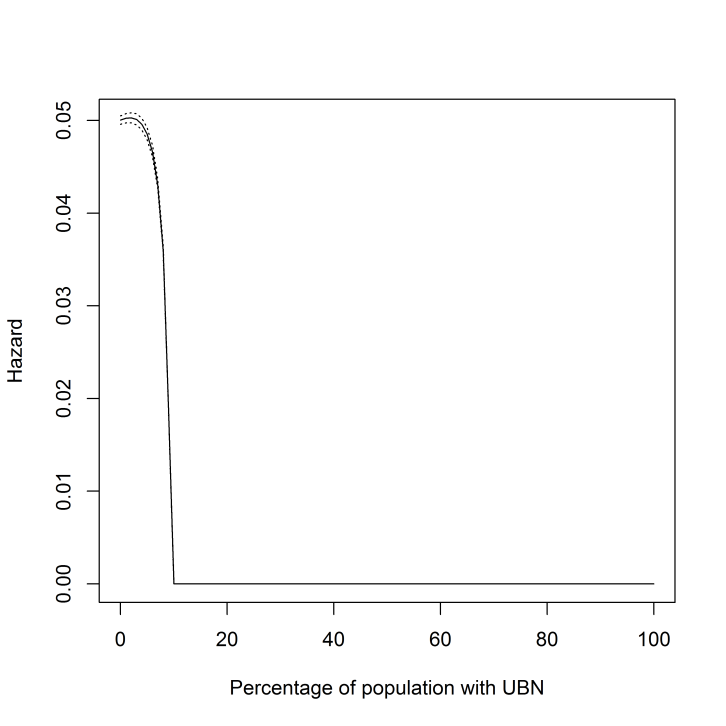 |
| --- | --- |

| D(i)  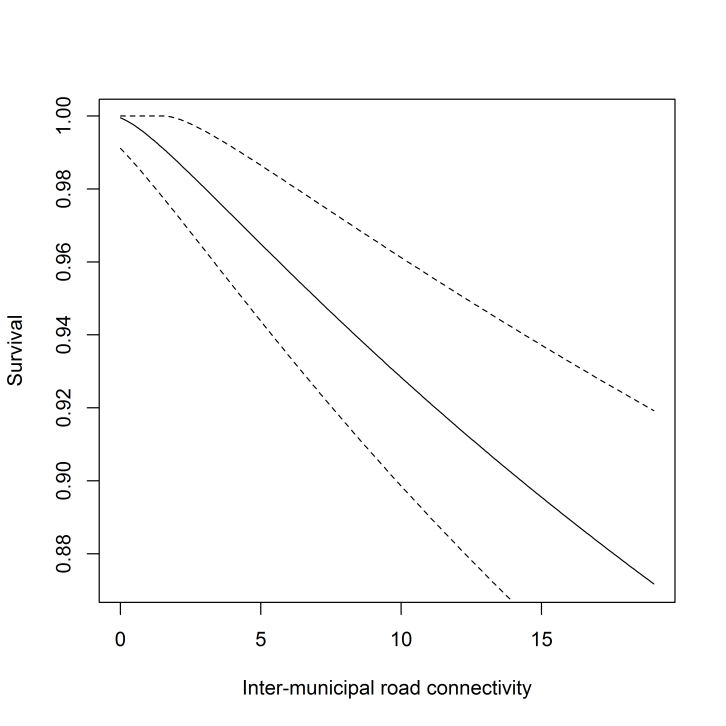 | (ii)  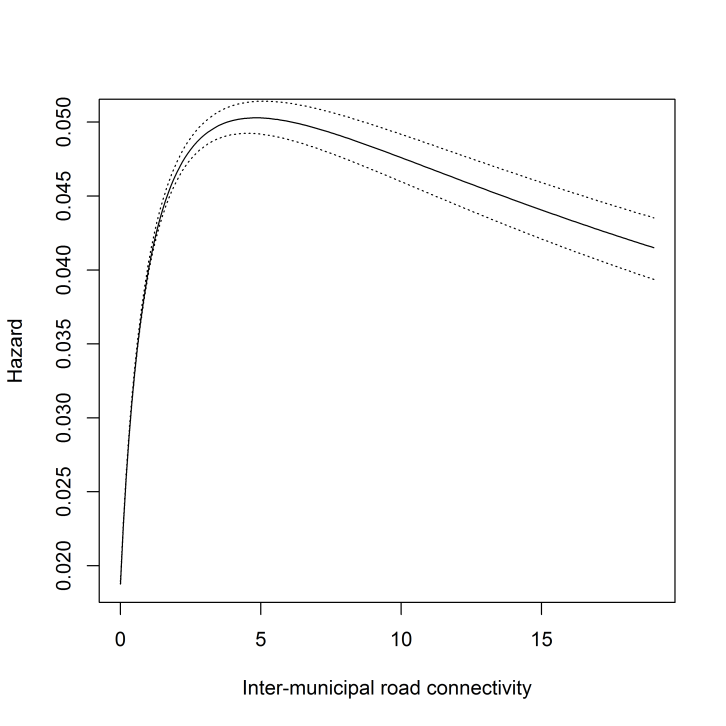 |
| --- | --- |

| F(i)  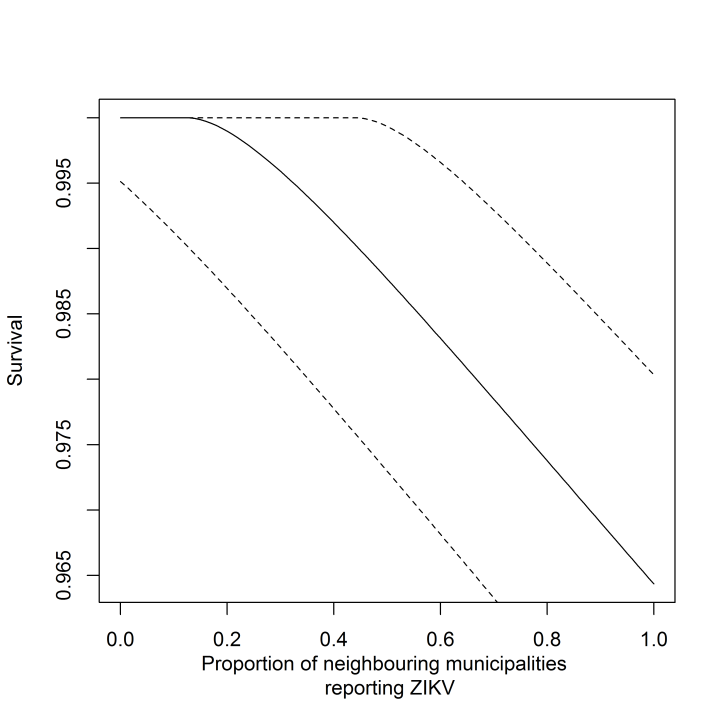 | (ii)  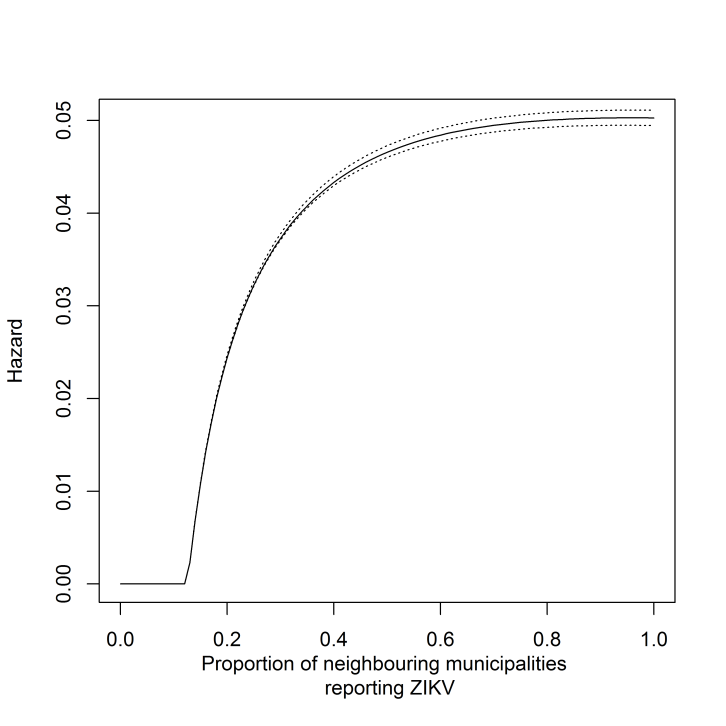 |
| --- | --- |

| G(i)  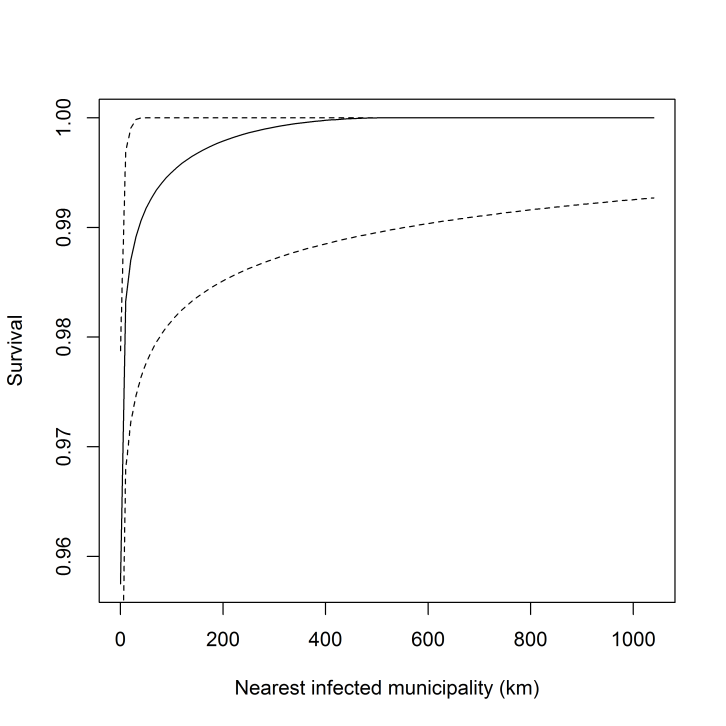 | (ii)  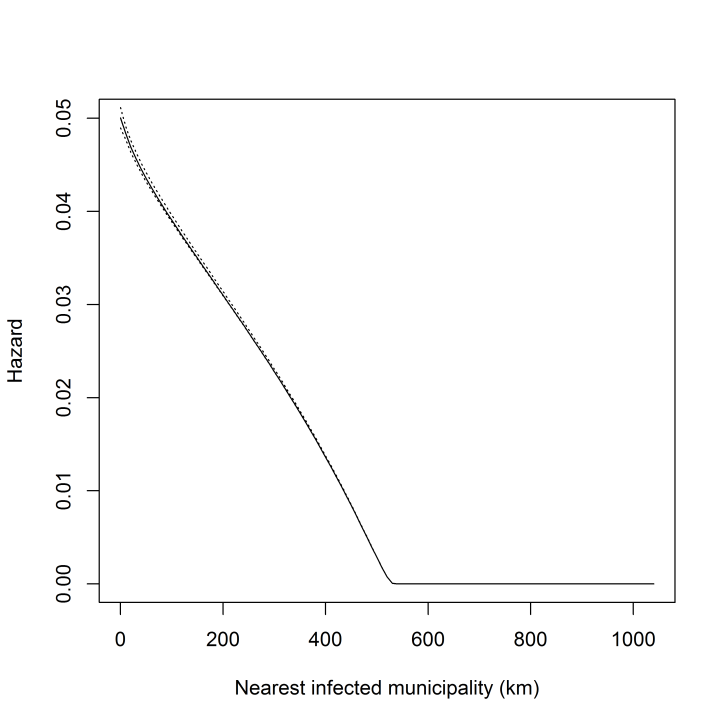 |
| --- | --- |

**Figure 3.2.c.** (i) survival and (ii) hazard functions given a) municipality elevation (m), b) total weekly precipitation (mm), c) UBN, d) inter-municipal connectivity, f) proportion of neighbouring municipalities reporting ZIKV, and g) nearest municipality reporting ZIKV (km).

**AF3.3 VARIABLE EFFECTS**

Using our best logistic and AFT models we used an AIC model selection approach to quantify the effects of the variables. Variables were assessed within groups as themed vector-related, socioeconomic-related and ZIKV neighbourhood disease intensity. We calculated the difference in AIC (∆AIC) for the best model against candidate models with an alternative variable from the same group theme. We also compared the best model to candidate models with no variable from the group theme. Models with a difference in AIC (∆AIC) < 2 from the best model provided effectively equal support [1].

For the logistic model, “Zika neighbourhood disease intensity” variables had the highest impact, as driven by the week of reporting. This variable is explicitly modelled to account for serial dependence in the outcome variable, so if we consider the next highest impact we see that the “vector-related” variables are highly important on influencing the probability of a first reported ZIKV case (ΔAIC 391.1). For the AFT model, time is implicitly accounted for in the modelling approach, and thus the impact of “Zika-spread” related variables is less because week of reporting is not a model variable. Like the logistic regression, it can be seen that “vector-related” variables are highly important on influencing the time to a first reported ZIKV case (ΔAIC 652.6).

**AF3.3.1 AIC model comparisons**

We found that accounting for the spatial distribution and temporal progression of known ZIKV cases (neighbourhood disease intensity variables) had the greatest impact on model fit (∆AIC = 3558.9; Table 3.2.1.a), followed by variables associated with vector abundance and ZIKV development in vectors (∆AIC = 391.1), and then lastly socioeconomic variables (∆AIC = 128.1). In assessing the impact of individual variables within a variable group, notable results for the vector-related group were that elevation, mean weekly temperature and mean weekly precipitation variables, even with one and two-week time lags, did not improve model fit beyond the null model (i.e. model excluding all variables in group). In the socioeconomic-related group, population density did not have an impact on improving model fit compared to the null model. Model fit was improved mostly by an interaction between UBN and inter-municipal road connectivity, followed by an interaction between UBN and road density. In the ZIKV-spread related group, accounting for reporting week had the greatest improvement over the null model, followed by the proportion of neighbouring municipalities reporting ZIKV, and then distance to nearest municipality reporting ZIKV (distance). Candidate models that have lower AIC values than the null model excluding all variables in the group are not shown in Table 3.3.1.a.

**Table 3.3.1.a.** ∆AIC between best and candidate models.

| **Variable group** | **Variable(s) used in model for variable group** | **AIC** | **∆AIC** |
| --- | --- | --- | --- |
| **Vector-related** | **Best model:** temperature + precipitation | 6183.5 | 0.0 |
|  | Temperature | 6217.1 | 33.7 |
|  | Vector index | 6393.6 | 210.1 |
|  | Model excluding all variables in group | 6574.5 | 391.1 |
| **Socioeconomic-related** | **Best model:** UBN + connectivity + UBN x connectivity | 6183.5 | 0.0 |
|  | UBN + road density + UBN x road density | 6214.5 | 31.0 |
|  | UBN | 6226.3 | 42.9 |
|  | Connectivity | 6279.3 | 95.8 |
|  | Road density | 6287.5 | 104.0 |
|  | Model excluding all variables in group | 6311.6 | 128.1 |
| **Zika neighbourhood disease intensity** | **Best model:** neighbours + week | 6183.5 | 0.0 |
|  | Week | 6203.8 | 20.3 |
|  | Neighbours | 8181.3 | 1997.8 |
|  | Distance | 8860.4 | 2676.9 |
|  | Model excluding all variables in group | 9742.3 | 3558.9 |

temperature = mean study period nighttime temperature

precipitation = total study period precipitation

neighbour = proportion of neighbouring municipalities reporting ZIKV

distance = distance to nearest municipality reporting ZIKV

week = week of reporting

Slightly different from the logistic regression model, we found that accounting for vector abundance and ZIKV development in vectors had the greatest impact on model fit (∆AIC = 652.2; Table 3.3.1.b), rather than the neighbourhood disease intensity factors (∆AIC = 326.7), but then as with the logistic regression model the socioeconomic variables had the least impact (∆AIC = 185.8). In the vector-related group a model with mean weekly nighttime temperature fit better than using mean elevation, however, the resulting model violated the assumption of data fitting to the parametric lognormal distribution. Candidate models that have lower AIC values than the null model excluding all variables in the group are not shown in Table 3.3.1.b.

**Table 3.3.1.b.** ∆AIC between best and candidate models.

| **Variable group** | **Variable(s) used in model for variable group** | **AIC** | **∆AIC** |
| --- | --- | --- | --- |
| **Vector-related** | **Best model:** Mean elevation + total weekly precipitation | 5428.9 | 0.0 |
|  | Mean elevation | 5698.4 | 269.4 |
|  | Total weekly precipitation | 5860.1 | 431.2 |
|  | Total weekly precipitation (t-1) | 5865.0 | 436.0 |
|  | Vector index | 5869.5 | 440.6 |
|  | Total weekly precipitation (t-2) | 5908.6 | 479.7 |
|  | Total study period precipitation | 6076.3 | 647.3 |
|  | Model excluding all variables representing hypothesis | 6081.6 | 652.6 |
| **Socioeconomic-related** | **Best model**: UBN + connectivity + UBN x connectivity | 5428.9 | 0.0 |
|  | UBN + connectivity | 5435.7 | 6.8 |
|  | UBN | 5515.2 | 86.3 |
|  | Connectivity | 5532.7 | 103.8 |
|  | Road density | 5604.7 | 175.7 |
|  | Population density | 5613.0 | 184.1 |
|  | Model excluding all variables representing hypothesis | 5614.8 | 185.8 |
| **Zika neighbourhood disease intensity** | **Best model**: Proportion of neighbours reporting ZIKV + distance to nearest municipality reporting ZIKV | 5428.9 | 0.0 |
|  | Proportion of neighbours reporting ZIKV | 5449.1 | 20.2 |
|  | Distance to nearest municipality reporting ZIKV | 5537.9 | 109.0 |
|  | Model excluding all variables representing hypothesis | 5755.7 | 326.7 |

**AF3.4 MODEL VALIDATION**

To validate the AFT model we could not use an approach of comparing predicted data to observed data. This is because the model assumes all municipalities can become infected, and thus, predicts the survival time at each time *t*, which is not translatable to observations of reporting ZIKV or not, at time *t*. Also, the eha R package ([*www.r-project.org*](http://www.r-project.org)) that we used to build the AFT model does not currently offer functions for model validation. Therefore to validate our model, we assessed the goodness of fit by visually assessing that the residuals fit the baseline distribution, as is typically done for AFT models [2]. We found that the distribution of residuals fit well with the log-normal distribution. We also used 10-fold cross-validation to assess model accuracy. In this approach we randomly divided the municipalities into 10 equal subsets. We could not equally subset the data because we needed to preserve the weekly sequence of observations at the municipality level. Then, following the 10-fold cross-validation methodology, we proceeded to train a model on K-1 groups, and then validate the model with the holdout group. We repeated this process 10 times ensuring that a different holdout group was defined each time. For each K data groupings, we calculated the mean of $t=e^{x_{i}^{'}\beta} \tau, t>0$ for both the training and the validation data, quantified the error as the difference between their means, and then calculated the overall error as the mean for all 10 errors (Table SM2.3.a). The overall error of -0.01 provides evidence that the model accuracy is high.

**Table 3.4.a** 10-fold cross-validation folds for training and validation data and their mean predicted $t=e^{x_{i}^{'}\beta} \tau, t>0$ and difference (i.e. error) between their values.

| Test | Training datasets | Validation dataset | Mean of predicted $t=e^{x_{i}^{'}\beta} \tau, t>0$  tor training data | Mean of predicted $t=e^{x_{i}^{'}\beta} \tau, t>0$  tor validation data | Error |
| --- | --- | --- | --- | --- | --- |
| 1 | 2 to 10 | 1 | 1.33 | 1.42 | -0.09 |
| 2 | 1, 3 to 10 | 2 | 1.35 | 1.45 | -0.10 |
| 3 | 1, 2, 4 to 10 | 3 | 1.39 | 1.32 | 0.06 |
| 4 | 1 to 3, 5 to 10 | 4 | 1.33 | 1.41 | -0.08 |
| 5 | 1 to 4, 6 to 10 | 5 | 1.45 | 1.53 | -0.08 |
| 6 | 1 to 5, 7 to 10 | 6 | 1.33 | 1.35 | -0.02 |
| 7 | 1 to 6, 8 to 10 | 7 | 1.28 | 1.28 | 0.00 |
| 8 | 1 to 7, 9, 10 | 8 | 1.43 | 1.48 | -0.04 |
| 9 | 1 to 8, 10 | 9 | 1.35 | 1.31 | 0.03 |
| 10 | 1 to 9 | 10 | 1.39 | 1.15 | 0.24 |
| **Overall error** | | | | | **-0.01** |

**AF3.5 REFERENCES**

1. Burnham KP, Anderson DR. Model Selection and Multimodel Inference: A Practical Information-Theoretic Approach. 2nd Ed. New York, USA: Springer-Verlag; 2002.

2. Galanova NS, Lemeshko BY, Chimitova E V. Using nonparametric goodness-of-fit tests to validate accelerated failure time models. Optoelectron Instrum Data Process. 2012;48: 580–592. doi:10.3103/S8756699012060064
